# Hyperspectral Imaging for the Colorimetric Characterization of Purple Manuscripts: Accuracy, Biases, and Diagnostic Potential

**DOI:** 10.3390/s26113358

**Published:** 2026-05-26

**Authors:** Cristina Fornacelli, Costanza Cucci, Andrea Casini, Maurizio Aceto, Marcello Picollo

**Affiliations:** 1 Institute of Applied Physics “Nello Carrara” (IFAC), National Research Council (CNR), Via Madonna del Piano 10, Sesto Fiorentino, 50019 Florence, Italy; c.cucci@ifac.cnr.it (C.C.);; 2 Dipartimento per lo Sviluppo Sostenibile e la Transizione, Università degli Studi del Piemonte Orientale Ecologica, Piazza S. Eusebio 5, 13100 Vercelli, Italy; maurizio.aceto@uniupo.it

**Keywords:** hyperspectral imaging, colorimetry, CIE Lab*, Specim IQ, purple codices

## Abstract

Color measurement and monitoring of chromatic changes over time play a key role in the study and conservation of historical materials. In this context, hyper-spectral imaging (HSI) offers spatially resolved spectral information that can be converted into colorimetric data, although its quantitative reliability under in situ conditions remains challenging. This study evaluates the colorimetric performance of a HSI system (Specim IQ) through comparison with a reference spectrocolorimeter (Konica-Minolta CM-700d), combining laboratory measurements on certified standards and in situ analyses on purple-dyed manuscripts. Colorimetric coordinates (CIELAB) and color differences (ΔE_00_) were used to assess accuracy, precision, and systematic deviations. Under controlled conditions, HSI showed good agreement with reference measurements, although systematic biases were observed. In situ applications revealed reduced accuracy (average ΔE_00_ ≈ 4.3) due to material heterogeneity and acquisition constraints. Despite these limitations, HSI preserved consistent relative chromatic relationships, enabling meaningful comparative analysis. Spatially resolved mapping of colorimetric parameters proved effective for visualizing chromatic variability, dye distribution, and degradation patterns. These results demonstrate that, while not fully reliable for absolute colorimetric assessment in situ, HSI represents a powerful tool for non-invasive, spatially resolved color analysis of complex historical materials.

## 1. Introduction

### 1.1. Hyperspectral Imaging for Colorimetric Applications

Color represents a key factor for quality assessment across a wide range of fields, including food science, industrial production, and cultural heritage [1,2,3]. In conservation science, color analysis has progressively evolved from a purely descriptive approach to a quantitative tool supporting conservation, restoration, and monitoring of artworks. Indeed, the colorimetric properties of a surface, and their variation over time, can provide essential information for the identification and characterization of materials, the detection of chromatic alterations, the definition of appropriate conservation strategies, and the early diagnosis of degradation processes [4,5,6,7,8].

Color perception arises from the combined effect of optical and psycho-physical processes, including the spectral power distribution of the illuminating source, the interaction of radiation with matter, and the response of the human visual system [9,10]. Within this framework, the study of color in cultural heritage has traditionally relied on spectrophotometric and colorimetric techniques, which provide quantitative descriptors of reflectance and enable the calculation of color coordinates in standardised spaces (e.g., CIE Lab*). However, while robust and widely adopted, traditional techniques present intrinsic limitations. In particular, the relatively large spot size (typically 3–8 mm in diameter) and the requirement for contact (or close proximity) make colorimeters poorly suited for highly heterogeneous and delicate surfaces, as it is the case for many polychrome artefacts. In such cases, spot analytical techniques are commonly used to establish reference conditions and to evaluate chromatic changes induced by ageing, environmental factors, or conservation treatments [3,11]. However, these methods are unsuitable to capture the spatial complexity and color variability that characterize many cultural heritage materials. As a result, their application is often confined to specific contexts, such as monitoring conservation processes or evaluating material stability, rather than providing a comprehensive characterization of chromatic heterogeneity.

In this context, hyperspectral imaging (HSI) has emerged as a powerful non-invasive technique for the analysis and documentation of cultural heritage materials [12,13,14,15]. Recent developments in portable systems [16,17], combined with advances in detector technology and data processing, have enabled the acquisition of calibrated reflectance data across the visible and near-infrared range with high spectral and spatial resolution. In accordance with CIE recommendation [18,19,20,21], the access to full reflectance spectrum at each pixel offers a robust dataset for color analysis, also enabling spatially resolved color measurements under standardized illuminants and geometries [22,23]. These features would make HSI particularly suitable for a comprehensive documentation of chromatic variability over extended surfaces, as well as for the monitoring of color changes over time [24]. Nevertheless, the extraction of accurate and reproducible colorimetric information from HSI data remains challenging. The colorimetric reliability of HSI systems depends on multiple instrumental and experimental factors, including the spectral power distribution and stability of the illumination source, the spectral sensitivity and linearity of the detector, the geometrical configuration of the acquisition setup, and the accuracy of white and dark reference calibration [22,24]. All these issues are even more relevant when operating in situ, where experimental conditions are inherently variable—and often far from ideal—and cannot be properly controlled, making the acquisition conditions and experimental protocols particularly difficult to standardise. Indeed, differences in environmental lighting (e.g., indoor versus outdoor conditions, or the impossibility to fully suppress residual ambient illumination), constraints in working distance and accessibility, as well as surface heterogeneity and non-planarity introduce significant variability in the acquired data. As highlighted by Cherubini et al. [24], these factors can strongly affect measurement reproducibility, limiting the comparability of datasets acquired under different conditions. Consequently, a lack of robust and transferable validation strategies remains a major limitation for the widespread use of HSI in quantitative colorimetric studies.

Specific analytical challenges emerged from an extensive investigation of purple codices carried out within the PRIN 2020 *‘Purple Parchment Legacy’* Project (https://purpleproject.it/ (accessed on 1 May 2026)). Purple codices represent a particularly complex class of manuscripts, in which color plays a central role both in technological interpretation and conservation issues. The purple coloration of parchment, typically obtained using organic dyes such as orchil (*Roccella* spp.) and folium (*Chrozophora tinctoria*) [25], is characterized by a high degree of variability, influenced by the use of different raw materials, extraction methods, application techniques, and substrate properties [26] (Figures S1 and S2). Furthermore, these dyes are highly sensitive to light and environmental conditions [27], thus undergoing progressive fading and significant chromatic alterations over time. Such a susceptibility results in highly heterogeneous chromatic values, often within the same page, making point-based colorimetric measurements inadequate to capture the full complexity of the system.

The use of HSI technique for providing a 2D colorimetric analysis has been therefore explored. Although previous studies have addressed the feasibility of hyperspectral imaging for colorimetric applications in various contexts, the specific challenges posed by purple codices require a dedicated and application-oriented approach. In particular, the variability of HSI-derived colorimetric data as a function of experimental setup necessitates a systematic assessment of measurement reliability under both controlled and real conditions.

### 1.2. Factors Limiting Colorimetric Accuracy in HSI

Previous studies investigating the colorimetric performance of multi- and hyperspectral cameras have emphasized several methodological constraints that require careful consideration [24,28].

In HSI acquisitions based on push-broom systems, inherent geometric factors may introduce slight illumination gradients across the scanned surface [29]. Conversely, reduced detector sensitivity and signal-to-noise ratio in the blue region (<450 nm) can affect the tolerance of b* estimation, particularly for highly reflective yellow and orange standards. Moreover, CMOS detectors, commonly used in portable HSI systems, can approach intensity saturation in high-intensity regions, potentially influencing L* estimation and reducing spectral detail [30,31].

In addition, glare and stray light effects can result in relevant optical artifacts, as they introduce additional contributions into the recorded reflectance spectra [32]. This effect becomes more critical in high-contrast scenarios, such as in simultaneous white calibration on darker backgrounds, where glare may be amplified. In push-broom hyperspectral systems, stray light may arise from internal scattering within the spectrograph or from imperfect optical isolation between adjacent pixels, leading to subtle spectral alteration. Although calibration procedures are designed to mitigate such effects, residual stray light cannot be entirely excluded and may contribute to systematic biases in derived colorimetric parameters.

In contrast to intrinsic system-related factors, acquisition-related variables can also significantly influence the reliability of the results. Therefore, an optimized acquisition strategy—carefully considering background selection, calibration mode, measurement distance, and illumination conditions—is essential to enhance reproducibility and minimize inter-instrumental variability.

### 1.3. Purple Codices and Analytical Challenges

Since Antiquity, purple has been associated with prestige and high social status [33,34]. Between Late Antiquity and the early Middle Ages (5th–11th century, with earlier evidence dating back to the 3rd–4th century), the application of purple dyes to parchment led to the production of the so-called *codices purpurei* [26,35].

Historical sources provide limited and often ambiguous information on the dyes used, as the term “purple” generally refers to a chromatic category rather than a specific material. Recent studies have identified orchil and folium as the main dyes used in parchment coloring, although their historical use and chronology remain difficult to reconstruct due to terminological ambiguity [36].

Orchil was obtained by various lichen species—including *Roccella tinctoria*, *R. fuciformis*, *R. phycopsis*, *Ochrolechia parella* or *Lasallia pustulata*. The hue of orchil-based dyes can widely change from bluish-red, to brownish-red up to intense purple depending on several factors—such as the lichen species, ammonia concentration and its ratio to lichen, maceration time and conditions, and dyeing technique [26].

In the Western world, the use of orchil significantly declined during Late Antiquity, before being rediscovered from the thirteenth century onward [37]. During the early Middle Ages, orchil was progressively replaced by *folium*, a dye derived from *Chrozophora tinctoria* (L.) *A. Juss* (also known as turnsole or *morella*). Blue and purple hues are specifically related to different stages of fruit maturation at the time of dye extraction [36,38].

A discrimination between orchil and folium—especially considering only non-invasive analytical methods—is still challenging [36]. In reflectance-based techniques, such as FORS and HSI, orchil and folium are characterized by broad absorption features in the visible region, typically resolved into two sub-bands. Folium is generally characterized by a slight shift toward shorter wavelengths (≈540–575 nm), whereas orchil shows maxima at longer wavelengths (≈545–595 nm) [39]. However, these features can be strongly influenced by pH, redox conditions, and substrate effects, often resulting in partial spectral overlap, which can significantly hinder reliable discrimination between the two dyes.

In this context, the complementary use of other complementary techniques, including colorimetric analysis, can provide additional evidence to support dye identification, enhancing the interpretation of spectral data.

Similarly, orchil and folium exhibit comparable features in the IR region, with a broad band in the 1650–1600 cm^−1^ range, where aromatic C=C contributions may overlap with the dominant amide I band of the collagen substrate and water-related absorptions. Additional bands in the 1200–1000 cm^−1^ region are mainly associated with polysaccharide components (C–O and C–O–C stretching), but are often masked by contributions from the support and binding media [39]. Raman spectroscopy is frequently limited by fluorescence, while even advanced mass spectrometry can be hindered by degradation products and the lack of specific molecular markers. Consequently, reliable differentiation requires an integrated multi-analytical approach supported by historical and technological context.

When applied on parchment, the coloring of purple dyes is also strongly influenced by the nature of the substrate. The chromatic differences between grain and flesh side of parchment represent a well-documented feature arising from the intrinsic structural anisotropy of animal skin and its transformation during manufacture [26,35,40]. These differences are often still perceptible in finished parchment, both in terms of texture and optical appearance, with the two surfaces showing variable degrees of smoothness, reflectance, and coloration [26,41,42].

The materials and treatments involved in parchment manufacture and surface preparation play a crucial role in determining its optical and colorimetric response. As highlighted by Vnouček et al. [43], the final properties of Late Antique parchment are largely established during the wet processing stages, including liming, dehairing, stretching, and peeling of the epidermal layer, which result in a very thin, smooth, and compact surface. These processes significantly influence surface roughness, fibre organization, and internal scattering, thereby affecting the optical characteristic and the resulting light-interaction. At the same time, residual features such as fibrous textures on the flesh side, local variations in thickness, or the presence of surface residues (e.g., calcium-based compounds or conservation treatments) can introduce additional scattering and modify the apparent brightness and chromatic response.

Therefore, the colorimetric response of the colored parchment must be interpreted as the result of a complex interplay between the colorant and the intrinsic structural and compositional features imparted by its manufacture and subsequent surface modifications.

From an optical and colorimetric perspective, such structural heterogeneity directly affects the interaction between the substrate and applied colorants. The denser grain side generally promotes a more superficial and uniform absorption of dyes, resulting in higher chromatic saturation, while the more porous flesh side favors deeper penetration and increased light scattering, leading to a lighter and less saturated appearance [26,43]. These inherent differences are further modulated by parchment manufacturing processes—such as liming, scraping, and surface finishing—which may accentuate or mitigate the contrast between the two sides. As a result, the distinction between hair and flesh sides constitutes not only a technological marker, but also a significant source of variability in colorimetric measurements. In this context, colorimetric analysis represents a valuable tool for monitoring chromatic features and supporting the non-invasive study of ancient dyeing materials and techniques.

### 1.4. Aim of the Study

The present study aims at evaluating the feasibility and reliability of hyperspectral imaging (HSI) for the specific task of colorimetric analysis of complex and heterogeneous historical materials, with specific application to purple-dyed parchment. A combined methodological approach, integrating controlled laboratory experiments and in situ measurements on historical manuscripts, is adopted to systematically assess the influence of instrumental factors (e.g., illumination conditions, acquisition geometry, and calibration procedures) and application-related variables (e.g., surface heterogeneity, material composition, and structural features of parchment) on the reliability of the colorimetric information derived from HSI data.

A validation strategy based on the direct comparison between HSI-derived colorimetric parameters and reference spectro-colorimetric measurements is implemented, allowing for the evaluation of inter-instrument differences and the identification of systematic biases under different acquisition conditions.

In addition, the study investigates the capability of HSI to preserve meaningful chromatic relationships under non-controlled conditions, supporting its use for comparative color analysis. The proposed methodology is finally applied to a selection of ten purple codices, demonstrating the potential of HSI as a tool for advanced colorimetric investigation and spatially resolved documentation of complex cultural heritage materials.

## 2. Materials and Methods

### 2.1. Samples

The study is structured around three datasets, each addressing a different methodological objective, namely Set 1, Set 2 and Set 3. In situ analyses were carried out on a selection of ten purple codices produced between Late Antiquity and the Renaissance (Table 1). The selected case studies constitute a representative sample of the variability typically encountered in purple-dyed manuscripts and provide a particularly challenging context for evaluating the performance of HSI under real cultural heritage conditions.

The investigations carried out as part of the ‘Purple Parchment Legacy’ Project aimed at providing a comprehensive characterization of the materials and techniques employed in the production of purple manuscripts throughout the centuries, spanning from Late Antiquity to the Modern Era. In this framework, the codices have been preliminarily investigated using complementary spectroscopic analytical techniques, such as Fiber Optic Reflectance Spectroscopy (FORS), allowing independent identification of the dyeing materials and supporting the interpretation of the chromatic data.

An overview of the three datasets considered in this study is provided below.

Set 1 (Laboratory dataset): Eight certified Diffuse Reflectance Spectralon^®^ color standards (Labsphere, New Hampshire, USA)—nominally red, orange, yellow, green, cyan, blue, violet, and purple—measured under controlled conditions using both HSI and a spectrocolorimeter, for method validation and setup optimization (Table S1 in Supplementary Materials). The Spectralon^®^ standards, with a diameter of 3 cm, are characterized by stable and well-defined reflectance properties across the visible range, making them suitable as reference materials for colorimetric evaluation and inter-instrument comparison.

Set 2 (In situ validation dataset): 27 selected areas from four representative purple codices (*Codex Neapolitanus*, *Codex Purpureus Sarzanensis*, *Durazzo Book of Hours* and the *Evangeliarium*) colored with orchil-based dyes as confirmed by preliminary FORS measurements. The areas were measured using both HSI and spectrocolorimetry, to assess inter-instrument agreement under real conditions. The codices were selected in order to reliably represent the variability observed in purple-dyed manuscripts (e.g., different parchment quality, color intensity, states of conservation and period).

Set 3 (In situ dataset): A larger dataset acquired on 86 areas across ten purple codices colored by orchil- and/or folium-based dyes. Colorimetric analysis of these areas was performed only with HSI, with the specific aim of investigating chromatic variability both within the same page (dye) and/or across different dyeing materials.

For Sets 2 and 3, the nature of the purple dye was previously investigated by FORS (see technical details in the next section).

### 2.2. Experimental Set Up

The colorimetric study was performed by both conventional colorimeter and portable HSI system. A Konica Minolta spectro-colorimeter (model CM-700d, Konica Minolta Sensing, Inc., Osaka, Japan) was used for reference color measurements. The instrument is equipped with an integrating sphere and operates with a diffuse/8° geometry (d/8°), enabling measurements with or without the specular component. In the present study, only the diffuse reflected radiation (Specular Component Excluded, SCE, mode) was considered, as the HSI system acquisition is primarily supposed to capture diffusely reflected radiation with the adopted geometry setup. The use of SCE mode thus ensures greater consistency and comparability between the two instruments.

The measurements were performed with 8 mm diameter spot accessory (MAV configuration). The CM-700d operates in the 400–700 nm spectral range with a 10 nm acquisition interval and utilizes a pulsed xenon lamp as the light source (with a UV cut filter) and a silicon photodiode array as the detector. Calibration was performed using the white reference tile (100% reflectance) and black trap (0% reflectance) provided by the manufacturer.

The HSI data were acquired using a portable push-broom hyperspectral imaging system Specim^®^ IQ (Specim, Spectral Imaging Ltd., Oulu, Finland), operating in the 400–1000 nm spectral range with a mean spectral resolution of 7 nm across 204 bands. The device combines a spectral camera, internal scanning mirror, embedded processing unit, and touchscreen interface in a compact module (207 × 91 × 74 mm, 1.3 kg). Being a line-scanning system, the Specim IQ captures hyperspectral data cubes through internal frame-shift scanning without the need for external moving stages. Each acquisition yields a data cube—spatial dimensions (512 × 512 pixels) by spectral dimension (204 bands)—providing a reflectance spectrum per each pixel of the imaged area.

Prior to every acquisition session, the system was calibrated using a 99% reflectance Spectralon^®^ (Labsphere, Sutton, NH, USA) white reference panel and following different manufacturer protocols, namely custom (CW) and simultaneous (SW) modes. Simultaneous white (SW) was acquired within the scene, while custom white (CW) was recorded separately under controlled conditions prior to measurements. The Spectralon^®^ white reference panel was captured under the same illumination conditions. Dark current correction was applied via the camera’s internal shutter.

All measurements were performed in a darkened room. Illumination was provided by two 50 W low-voltage tungsten halogen lamps (HALOSTAR ST, 12 V, OSRAM GmbH, Munich, Germany), characterized by a color temperature of approximately 2950 K and a color rendering index Ra = 100 and equipped with a UV filter to reduce potentially harmful ultraviolet radiation. The lamps were allowed to warm up and stabilize to ensure consistent output. A 2 × 45°/0° geometry was adopted to provide uniform lighting, while minimizing specular reflections to avoid direct irradiation and limit localized heating.

Since they represent a long-established reference in colorimetric measurements, halogen lamps were selected due to their continuous spectral power distribution across the VIS–NIR range, ensuring accurate hyperspectral reflectance acquisition and colorimetric calculations [44]. In contrast, LED sources may exhibit spectral irregularities, particularly in the near-infrared region, potentially affecting reflectance reconstruction [45]. Although LED-based illuminants can be engineered to approximate standard spectra, they require complex optimization and may still show residual spectral mismatch [46]. Xenon lamps were excluded due to their strong ultraviolet emission and intense spectral features in the near-infrared region, which may compromise hyperspectral data.

During in situ analysis, exposure time was carefully evaluated to ensure minimal light exposure and thermal impact in order to prevent any risk of damage to the parchment during measurements [47]. The manuscript was kept covered between successive acquisitions and exposed only for the time strictly required for the acquisition of each individual folio (typically less than 90 s).

Preliminary FORS investigations were performed to support the identification of orchil- and folium-based dyes. Measurements were performed via two Zeiss (ZEISS, Jena, Germany) spectroanalyzers, an MCS 601 UV-NIR model operating in the 190–1015 nm range, and an MCS 611 NIR 2.2 WR model operating in the 910–2200 nm range, with spectral resolution of 0.8 and 6.0 nm/pixel, respectively. The radiation source is a 10 W Zeiss CLH600 halogen lamp (ZEISS, Jena, Germany), with a correlated color temperature of approximately 3000 K and an emission spectrum spanning 320–2500 nm.

**Figure 1:**
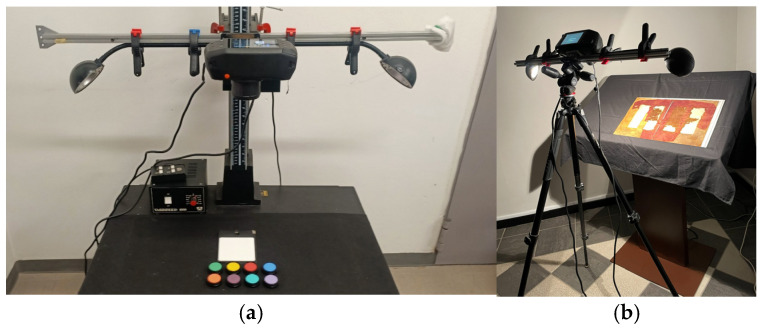
Experimental setups for hyperspectral imaging acquisition using a Specim IQ camera under controlled illumination in laboratory-based (**a**) and in situ (**b**) acquisitions.

ELSEC 764 UV+ (Littlemore Scientific Engineering, Gillingham, Dorset, UK) data logger was used to measure the amount of UV and visible radiation. To assess the homogeneity of the illumination field during acquisition, illuminance values across nine selected areas distributed over the entire surface were examined. The average illuminance at the target was 1370 ± 80 lux over the entire reference area (20 × 30 cm) and was considered sufficient for a good signal-to-noise ratio in the HSI data. For in situ measurements, a similar geometry was used, but intensity was intentionally lowered (~1000 l×) to minimize exposure of the sensitive manuscript to visible and IR radiation (Figure 1).

Acquisitions on Diffuse Reflectance Spectralon Standards (Set 1) were carried out under different acquisition conditions, varying the background color and calibration mode (Table 2). This test aimed to assess the influence of contrast conditions, particularly in the presence of adjacent high- or low-reflectance areas, and to determine optimal acquisition conditions for further in situ analysis.

### 2.3. Data Analysis and Image Processing

HSI reflectance spectra in the range 400–1000 nm were interpolated from 3.5 to 1 nm using a custom program developed at CNR-IFAC to calculate the CIE colorimetric values. The software calculates the colorimetric data for the 2° Standard Observer and D65 illuminant and returns grayscale TIFF format images for each of the three coordinates L*, a* and b*.

Fiji ImageJ software (ImageJ software package, U.S. National Institutes of Health, Bethesda, MD, USA) was used to extract colorimetric data in the CIELAB space [24]. Regions of interest (ROIs) were selected to extract colorimetric value from representative homogeneous areas, avoiding edges, damaged regions and zones affected by strong textural variations. ROI size of 15 × 15 pixels was selected to ensure adequate spatial resolution while minimizing the influence of local signal fluctuations and small-scale variability related to surface inhomogeneities.

Basic descriptive statistics were applied to the three datasets to assess data distribution, identify potential systematic biases, and evaluate overall measurement variability.

For Sets 1 and 2, CIELAB values from corresponding areas were also obtained using the Konica-Minolta CM-700d spectrocolorimeter (Konica Minolta, Inc., Tokyo, Japan). Inter-instrumental differences between the two analytical techniques were evaluated by calculating color coordinates variations, defined as follows:

ΔL*_inter_ = L*_HSI_ − L*_Col_

and similarly Δa*_inter_ and b*_inter_. Color differences (ΔE_00_) were then obtained using the CIEDE2000 formula [48].

While Sets 1 and 2 were used to evaluate the performance and reproducibility of HSI-derived colorimetric measurements, the analysis of Set 3 (in situ dataset) was based on a comparative approach. Given the reduced accuracy observed under real acquisition conditions, intra- and inter-codex comparisons were considered more representative than absolute colorimetric evaluation.

The high spatial resolution of HSI data enabled the generation of spatially resolved maps through mathematical transformations applied to grayscale TIFF images corresponding to the individual CIELAB coordinates (L*, a*, b*). In this framework, statistical analysis (Mann–Whitney test) was applied to evaluate the discriminating power of each chromatic coordinate; the results are reported and discussed in Section 4.3.

Threshold-based maps were generated by classifying pixel values according to ranges defined from the statistical distribution of reference datasets. Thresholds were determined using percentile-based criteria (e.g., quartiles), allowing segmentation into different chromatic domains. Binary masks corresponding to each class were generated, converted to 32-bit format, normalized, and combined through image algebra operations to produce classified maps. Consistent visualization was ensured by applying fixed lookup tables (LUTs) across all images.

Degradation phenomena were investigated through derived maps based on the combined analysis of a* and b* coordinates. Previous studies on orchil dyes [27,49] have shown that photodegradation affects the chromophore components responsible for the purple hue, leading to their progressive loss and to measurable chromatic shifts, typically characterized by a decrease in a* followed by an increase in b* values. This behaviour reflects a gradual transition from dye-dominated to substrate-dominated optical response. A similar chromatic trend can be assumed when the loss the of coloring material is not driven by chemical degradation, but by the physical removal of the dye layer, as in the case of mechanical abrasion.

Within this framework, two complementary empirical descriptors were introduced for the purposes of this study to enable the spatial mapping of color loss across the surface. Although not physically defined, these descriptors are intended to facilitate the visualization and interpretation of chromatic trends rather than to provide absolute quantitative measures.

The first descriptor, defined as (b* − a*), was designed to explore the local balance between the dye contribution (represented by a*) and that of the parchment substrate (represented by b*). It is based on a direct pixel-by-pixel combination of the color coordinates and is therefore sensitive to local variations across the surface, making it particularly suitable for mapping spatially heterogeneous effects, such as those induced by mechanical handling and abrasion. The descriptor thus allows the visualization of localized color loss and the assessment of wear patterns, highlighting intra-surface variability.

In contrast, the second descriptor, defined as (Δb* − Δa*), is based on deviations from reference values derived from well-preserved areas of the same manuscript. The descriptor therefore quantifies difference from a reference “initial” state, allowing the assessment of overall color change. The use of inter-codex reference areas is essential, as the initial chromatic properties may vary significantly between manuscripts, and even within the same folio (e.g., recto/verso or different gatherings). While spatial implementation of this descriptor still allows the visualization of local variations within the investigated area, photodegradation is expected to produce more uniform alterations across the folio resulting in less structured spatial patterns than those associated with localized processes such as mechanical abrasion.

Although both descriptors rely on the same underlying chromatic behavior—namely the decrease in a* and the increase in b* associated with dye loss—they differ in their interpretative meaning, with the former describing the spatial distribution of the current chromatic state, while the second highlights variations relative to a reference condition. The two approaches are thus complementary and were used to enhance the spatial visualization of degradation patterns.

All derived maps were interpreted as relative representations, as their outcome depends on the selected thresholds and reference values.

## 3. Results

### 3.1. Colorimetric Accuracy on Reference Samples (Set 1)

Set 1 was investigated via the Konica Minolta spectrocolorimeter and the Specim IQ hyperspectral camera, and the complete results are reported in Tables S1 and S2.

HSI results are organized according to the different acquisition modes described in the previous section—defined by the combination of background color and white reference mode. For each acquisition condition, mean values of ΔL*_inter_, Δa*_inter_, Δb*_inter_, and ΔE_00inter_ were calculated across the set of the eight color reference standards, providing an estimate of the average systematic deviation. This approach was preferred over a color-specific evaluation, as the aim of the study was not to assess the accuracy for individual hues, but rather to determine the overall performance of the acquisition setup. Although color-dependent differences cannot be excluded, the evaluation of average colorimetric accuracy across multiple reference standards was considered more representative of real conditions, where surfaces of artworks or historical objects are typically heterogeneous and polychrome. Indeed, in reflectance measurements, the recorded signal may be influenced not only by the intrinsic spectral properties of individual colors, but also by the local environment contrast conditions, determined by the adjacent high- and low-reflectance areas, potentially giving rise to glare or halo effects. Even when not visually evident, such effects may still contribute to overall measurement uncertainty.

Additional descriptive statistics (median, minimum, and maximum values) and root-mean-square error (RMSE) were calculated to further characterize the distribution and variability of the observed differences, and the results are summarized in Table 3.

As can be observed, the experimental conditions—particularly the choice of background color and the white reference mode—significantly influenced the colorimetric results.

In general, ΔL*_inter_ showed a systematic positive bias with good precision (SD < 1), although RMSE values consistently exceeded 1.15, indicating persistent systematic deviations. Similarly, Δa*_inter_ showed a slight positive bias, with high accuracy and precision (both SD and RMSE below 1 in most of the cases). In contrast, Δb*_inter_ was characterized by a marked negative bias and greater variability, except in B-CS and B-SM acquisition modes, where both SD and RMSE remained below 1. Across all the different set up conditions tested, yellow, red, and orange references showed the largest average ΔE_00inter_ values—primarily due to substantial Δb*_inter_ errors—except in acquisitions on a black background where the highest ΔE_00inter_ values were primarily associated with deviations in ΔL*_inter_ and Δa*_inter_.

To better assess the influence of the acquisition strategy, ΔE_00_ values were clustered according to commonly accepted perceptual thresholds [48,50]. The distribution of measurements across these thresholds (Table 4) enabled identification of the most reliable analytical configurations. The comparison between datasets is discussed below in relation to the main influencing factors:

White reference calibration: CS mode consistently produced more stable and accurate results than SM mode, reducing both perceptible and critical deviations. The distribution of average ΔE_00inter_ values clearly shows that simultaneous referencing shifts a substantial fraction of measurements toward higher perceptual categories, particularly in the 2 < ΔE_00inter_ ≤ 3.5 range.

Background: White and yellow backgrounds are associated with higher mean ΔE_00inter_ values (>2.10), mainly due to systematic biases in L* and b* components. In contrast, black and gray backgrounds ensure improved accuracy and repeatability. Although Gy-CM acquisition mode shows slightly lower mean and median ΔE_00inter_ values, B-CM is characterized by lower standard deviations, suggesting higher repeatability. Given the importance of consistency in colorimetric validation, the setup with lower dispersion may be preferred, even at the expense of a marginal increase in mean error.

SCI vs. SCE geometry: The differences observed when comparing HSI data with SCI/SCE measurements on diffuse reflectance standards are limited in magnitude. This can be explained considering that variations in accuracy between the two geometries are not necessarily attributable to the intrinsic colorimetric performance of the instruments, but rather to differences in the interaction with the physical and optical properties of the investigated surface. While this effect is negligible on ideal diffuse targets, it will be more relevant when measurements are performed on complex and heterogeneous substrates such as parchment, where micro-topography and spatially variable gloss influence the contribution of the specular component.

### 3.2. Purple Codices

Based on the results obtained under controlled laboratory conditions, CS mode was selected as the optimal calibration strategy for in situ measurements, due to its enhanced stability and reduced inter-instrumental variability.

HSI-derived colorimetric data obtained from Set 2 were compared with spectrocolorimetric measurements acquired in SCE mode, which was considered more suitable for a heterogeneous material such as parchment (Table 5). Indeed, the degraded parchment feature several micro-topography irregularities and occasional gloss that may cause surface-related artefacts. The exclusion of the specular component can help to reduce these effects improving the robustness of the comparison.

Compared to Set 1, the in situ inter-instrument comparison (Set 2) exhibited a clear reduction in both precision and accuracy, as evidenced by larger deviations in all three colorimetric coordinates. In particular, Δa*_inter_ (2.51 ± 1.50) and Δb*_inter_ (−2.51 ± 2.24) showed directional biases consistent with those observed under laboratory conditions, although with significantly increased magnitude and dispersion. In contrast, ΔL*_inter_ (−2.28 ± 3.16) revealed a systematic underestimation of lightness, accompanied by a high variability.

These deviations resulted in an average ΔE_00inter_ value of 4.32 ± 2.34 (RMSE = 3.90), indicating a substantial loss of colorimetric agreement under real acquisition conditions. The observed chromatic shifts can be attributed to the combined effects of local illumination variability, surface heterogeneity, and material degradation, which are inherent to in situ measurements that resulted difficult to control. The results show that only 10 areas (37% of the total) fall within the acceptable tolerance range (ΔE00_inter_ < 3.5), whereas 5 areas (19%) showed deviations exceeding 5.0. Notably, the largest discrepancies were consistently associated with parchment in advanced states of deterioration, as observed in SARZ.

Finally, colorimetric data obtained following the HSI investigation of Set 3 (Table 6) reveal a broad chromatic variability across the analyzed manuscripts, reflecting differences in material properties and conservation state.

The high variability of L* (22.06–60.68) can be mainly attributed to difficulties in achieving homogeneous illumination across the analyzed areas, together with substrate inhomogeneities. In contrast, a* (5.50–24.00) is consistently positive across all samples, indicating a dominant red component with moderate variability. The b* coordinate (−12.23–16.16) shows a pronounced dispersion reflecting shifts spanning both positive and negative values and reflecting shifts along the yellow–blue axis, from yellow-reddish (positive b*) to bluish (negative b*) tones.

Within this variability, orchil-based dyes are generally characterized by higher average a* values (14.29 ± 3.67) and positive b* values, with exceptions observed only forDUR where b* ranges between −8.40 and 0.77. In contrast, folium-based dyes show lower a* (9.23 ± 1.70) and predominantly negative b* values (−5.68 ± 3.99), consistent with a shift towards bluish hues. However, for a further discussion of these data, the systematic bias affecting the b* coordinate, as evidenced in Set 2, has to be taken into account.

## 4. Discussion

The results obtained both in laboratory-based and in situ conditions show systematic deviations (ΔL*_inter_ > 0, Δa*_inter_ > 0, Δb*_inter_ < 0) related to both instrumental and environmental factors that must be accounted for. The comparative analysis on Diffuse Reflectance Spectralon standards showed that these deviations were consistent across background conditions and measurement geometries (SCI/SCE), suggesting that they originate primarily from intrinsic characteristics of the hyperspectral system rather than from specific acquisition configurations. Therefore, the results also stress the need to adopt specific precautions in the acquisition setup to maximize the reproducibility of the results.

### 4.1. Sources of Systematic Bias in HSI Colorimetric Measurements

As observed under controlled laboratory conditions, both ΔL*_inter_ and Δa*_inter_ exhibited positive biases across all setups. These deviations can be attributed to a combination of factors, including the geometry of the push-broom acquisition system, illumination inhomogeneities, and glare-related effects.

In particular, specular reflections and stray light contributions may increase the recorded luminance, leading to an overestimation of L* and a reduction of local contrast. This interpretation is consistent with previous studies on the Specim IQ system [24,32] which studied the role of glare originating from stray-light within the internal optics of the spectrograph in the insurgence of spectral cross-contaminations between neighboring pixels and increased apparent brightness. In contrast, in situ measurements showed a systematic underestimation of L*, indicating that surface-related factors—such as non-planarity, irregular scattering and surface degradation—become dominant under real conditions. Additional contributions may arise from detector limitations in reflectance measurements, including potential saturation effects in highly illuminated areas.

The overestimation of a* might be further explained by the spectral power distribution (SPD) of the halogen lamp, which is characterized by enhanced emission in the long-wavelength region (>600 nm), potentially amplifying red-channel contributions. In contrast, Δb*_inter_ showed a greater variability and a marked underestimation, particularly for highly reflective areas such as yellow and orange standards. This behavior can be mainly related to the reduced emission of the halogen source and lower detector sensitivity in the blue region (400–500 nm), resulting in a lower signal-to-noise ratio.

**Figure 2:**
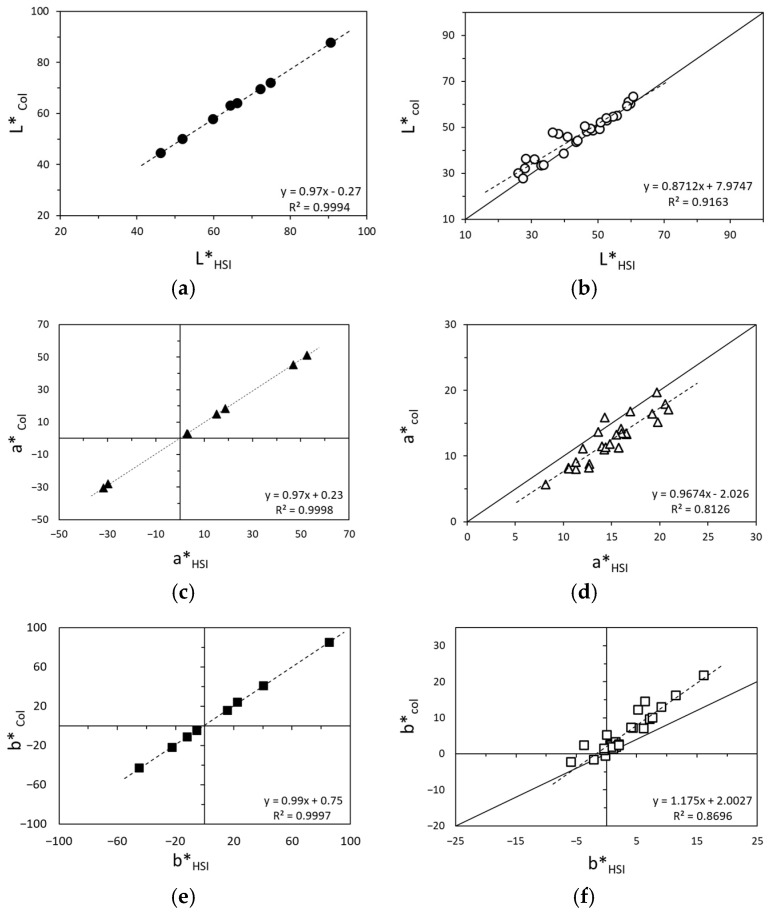
Comparison between HSI-derived and spectrocolorimetric (Konica Minolta CM-700d) color coordinates. (**a**,**c**,**e**) Laboratory measurements performed on reference standards (filled symbols) corresponding to set 1. (**b**,**d**,**f**) In situ measurements acquired on selected areas of purple codices (open symbols, corresponding to set 2. The dashed lines correspond to the linear regression model fitted to the data, whereas the solid line represents a reference line included to facilitate visual comparison between HSI-derived and spectrocolorimetric measurements.

In addition to the instrumental factors, acquisition setup parameters may also influence measurement stability. Background color was found to play a significant role, with black and gray backgrounds minimizing inter-reflection effects resulting in lower mean ΔE_00_ values with improved repeatability. Based on these results, the combination of black background and custom white reference calibration was identified as the optimal acquisition setup. Linear regression analysis performed under these conditions (Figure 2a,c,e) showed a strong agreement between HSI and spectrocolorimetric data for all coordinates (R^2^ ≥ 0.9994. For L* (m = 0.97; b = −0.27); the regression suggests a slight compression at higher reflectance levels, possibly due to detector saturation or glare effects.

A minor positive offset, likely related to residual spectral imbalance in the red region, was observed for a* (m = 0.97; b = +0.23), while b* (m = 0.99; b = +0.75) showed a slightly greater dispersion, consistent with increased spectral noise and reduced sensitivity in the blue region.

### 4.2. Limits and Potentialities of In Situ Applications

Despite the encouraging results obtained under controlled conditions, a critical understanding of the actual limits of HSI for in situ colorimetric applications is essential.

As discussed in the previous section, the colorimetric information extracted from HSI data cubes is not suitable for absolute comparisons, yet remains reliable and robust for relative color assessments. The anisotropic and fibrous nature of parchment causes heterogeneous reflectance, reducing measurement consistency. Degradation further alters optical properties, enhancing glare and specular reflections that can distort spectral data and affect colorimetric coordinates. Since HSI measurements are strongly dependent on acquisition conditions, it is therefore mandatory to understand how the texture of the samples and measurement conditions influence colorimetric evaluation.

While Set 1 showed a good linear agreement between HSI and spectrocolorimetric data, in situ acquisitions exhibit increased dispersion and reduced correlation. Set 2 revealed a clear inversion in the behavior of ΔL*_inter_, indicating a systematic underestimation of luminance by HSI under less controlled conditions. This behavior can be primarily attributed to surface non-planarity and material-related factors, including parchment warping, binding constraints, collagen deterioration, and, in more degraded areas, gelatinization and microcracking. However, the reduced light intensity used during in situ measurements to ensure the preservation of the manuscripts may also contribute to a lower signal reaching the sensor, further affecting luminance estimation.

In contrast, Δa*_inter_ and Δb*_inter_ followed trends consistent with laboratory observations, showing a systematic overestimation along the red–green axis and an underestimation along the yellow–blue axis. The relatively limited dispersion of Δa*_inter_ indicates a stable overestimation along the red–green axis, whereas the larger variability in Δb* confirms the reduced reliability of HSI measurements in the blue spectral region.

This variability, especially concerning L* values, strongly influences ΔE_00inter_ results (4.32 ± 2.34), confirming the limited absolute accuracy of HSI in non-controlled conditions.

The regression analysis (Figure 2b,d,f) shows a clear compression (m 0.87) and offset (b 7.97) of L*, consistent with luminance underestimation. The a* coordinate (m 0.97) preserves the positive trend observed in Set 1, but with increased dispersion (R2 0.81) and a systematic offset (b −2.03). In contrast, b* exhibits an apparent expansion (m 1.18) and higher variability, indicating lower reliability along the yellow–blue axis. Overall, although a linear relationship is maintained, it is affected by coordinate-dependent distortions and increased uncertainty under real conditions.

**Figure 3:**
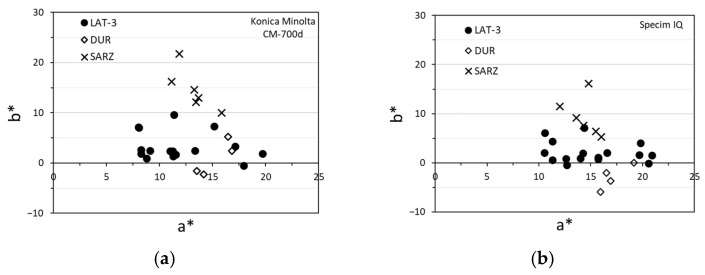
a* − b* biplot obtained from spectrocolorimetric (**a**) and HSI (**b**) measurements for selected folios of purple codices (Set 2).

Given the strong influence of acquisition geometry and surface properties on L* under in situ conditions, this parameter was not considered sufficiently reliable for subsequent chromatic comparisons. Consequently, the distribution of data in the a* − b* space was examined to assess the degree of agreement between HSI and spectrocolorimetric measurements. As expected, the a* − b* biplots (Figure 3) show a coherent spatial organization of the data, although a systematic shift on the a* axis is observed in the HSI dataset, together with a partial compression along the b* axis.

The compression along the b* axis is consistent with the systematic bias affecting the b* coordinate and can be interpreted as a reduction in chromatic dynamic range rather than a distortion of the underlying chromatic structure. Nevertheless, the spatial distribution of b* values remains meaningful and b*-based maps can be thus reliably used to investigate relative chromatic variations and identify patterns associated with material differences or degradation phenomena, provided that their interpretation is limited to intra-dataset comparisons.

In the following section, this approach is applied to the study of purple-dyed manuscripts, with the aim of exploring the chromatic signatures of different dyeing systems and assessing the potential of HSI for their discrimination.

### 4.3. Chromatic Characterization of Purple-Dyed Codices

Despite limitations affecting absolute colorimetric accuracy under in situ conditions, HSI has proven to be a powerful tool for comparative analysis, as it preserves consistent relative chromatic relationships between different areas. The stability of the relative variations in a* − b* space, as observed in the previous sections, allows the identification of chromatic trends associated with key parameters, such as colorant nature, application techniques, and/or degradation phenomena. In this context, the qualitative and spatially resolved nature of the colorimetric information derived from hyperspectral data represents a significant advantage for the study of complex systems such as purple-dyed codices. By providing spatially resolved chromatic variations across the imaged area, HSI provides valuable insights into material properties, technological aspects, and conservation-related phenomena, supporting a comprehensive interpretation of these valuable and fragile work of arts.

The following sections present selected application examples illustrating these capabilities, with results discussed through a progressively structured approach, moving from simpler cases to increasingly complex comparative scenarios.

#### 4.3.1. Dye-Substrate Interaction: Hair Side vs. Flesh Side

The results discussed in this section refer to a folio from the *Codex Neapolitanus* (LAT-3). This case represents the simplest analytical scenario, in which variability associated with raw material sources and extraction conditions is minimized, as the same dye (orchil) is examined within the same codex and fascicle. Under these conditions, the influence of the parchment substrate can be isolated and directly assessed in order to investigate how the different dye absorption behaviors depend on the side of the skin [26].

HSI data reveal a systematic chromatic shift between the grain (hair) and flesh sides, quantitatively expressed by a decrease in lightness (ΔL* = −8.23) and a marked increase in the red–magenta component (Δa* = +5.76), while variations along the b* axis remain limited (Δb* = +0.20); these trends resulted in a significant color difference (ΔE_00_ = 9.19).

This behavior could be likely interpreted in terms of the different microstructural and optical properties of the parchment [26,40,43]. The more compact grain side is expected to favor retention of the dye at the surface, thereby resulting in higher chromatic saturation and lower reflectance. In contrast, the more porous flesh side promotes deeper dye penetration and increased subsurface scattering, leading to lighter and less saturated appearances. The negligible variation in b* suggests that the observed difference is primarily driven by changes in dye concentration and distribution rather than by a shift in hue.

**Figure 4:**
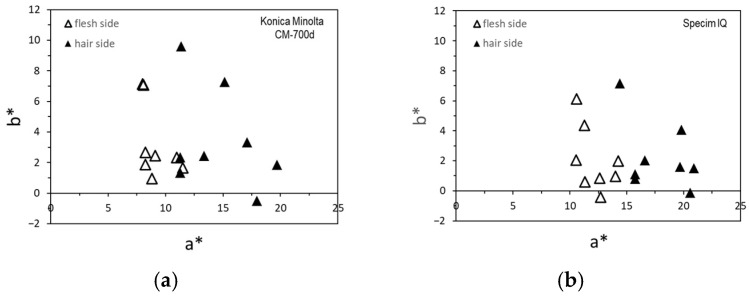
a* − b* biplots from selected areas (15 × 15 pixels) of flesh and hair sides of parchment dyed with orchil, obtained using a Konica Minolta CM-700d (**a**) and (Specim IQ), (**b**). Both datasets show a consistent separation between the two surfaces, with the hair side characterized by higher a* values. The agreement between the two measurement approaches confirms the ability of HSI to preserve relative chromatic relationships associated with the microstructural properties of the parchment.

As illustrated in Figure 4, the chromatic distinction between flesh and grain sides, particularly along the a* coordinate, shows strong agreement between HSI-derived data and the Konica Minolta color measurements, both in terms of trend and magnitude. This consistency supports the reliability of HSI for investigating chromatic variations related to substrate microstructure, while also demonstrating its added value in providing spatially resolved information.

#### 4.3.2. Discrimination Between Orchil and Folium

To quantitatively assess the chromatic differences between orchil- and folium-dyed samples, a non-parametric Mann–Whitney test was applied to the a* and b* coordinates obtained via HSI. The analysis was performed on orchil-dyed (LAT-3, DUR, GR-2, HERC) and folium-dyed (IB57, XIX27, XII34, ARTE) manuscripts and confirms the opportunity to obtain a clear and statistically robust differentiation between the two dyeing systems via HSI measurements.

As observed in Table 7, the Mann–Whitney test highlights a statistically significant difference between orchil- and folium-dyed samples for both chromatic coordinates (*p*-values < 0.0001), with large effect sizes (r = 0.63 for a+ and r = 0.66 for b*), indicating a strong and consistent separation between the two groups.

For the L* coordinate, folium samples exhibit higher median values (46.69) compared to orchil (38.60). The moderate effect size (r = −0.33) indicates that, although the difference in L* between orchil and folium samples is statistically significant (*p* = 0.0020), the magnitude of this difference is relatively limited. This suggests a partial overlap between the two groups and reflects the fact that lightness is influenced by multiple factors, including substrate properties and conservation state and illumination constrains, rather than being a direct indicator of dye composition. When the a* coordinate is considered, orchil samples show higher median values (14.12) compared to folium (9.36), suggesting a greater contribution of the red–magenta component in orchil-dyed areas. However, the interquartile range (IQR) is larger for orchil (4.96) than for folium (3.03), indicating higher chromatic variability within the orchil group. Variations along the a* axis contribute to the overall chromatic variability, but do not represent the principal axis of separation, as evidenced by the partial overlap of the confidence ellipses in this direction. This suggests that a* captures secondary chromatic differences, likely associated with variations in dye concentration, substrate interaction, or conservation state, rather than intrinsic differences between the two dyeing systems.

A more pronounced separation is observed for the b* coordinate, where orchil samples exhibit positive median values (1.32), while folium samples are clearly distributed in the negative domain (median = −6.38). This confirms that b* represents the primary chromatic parameter for discriminating between the two dyeing systems. The relatively broad IQR values for both groups (4.47 for orchil and 5.99 for folium) indicate some degree of overlap, likely related to local variability, degradation processes, and substrate influence. The role of the bias observed for b* values obtained via HSI have also to be taken into account.

**Figure 5:**
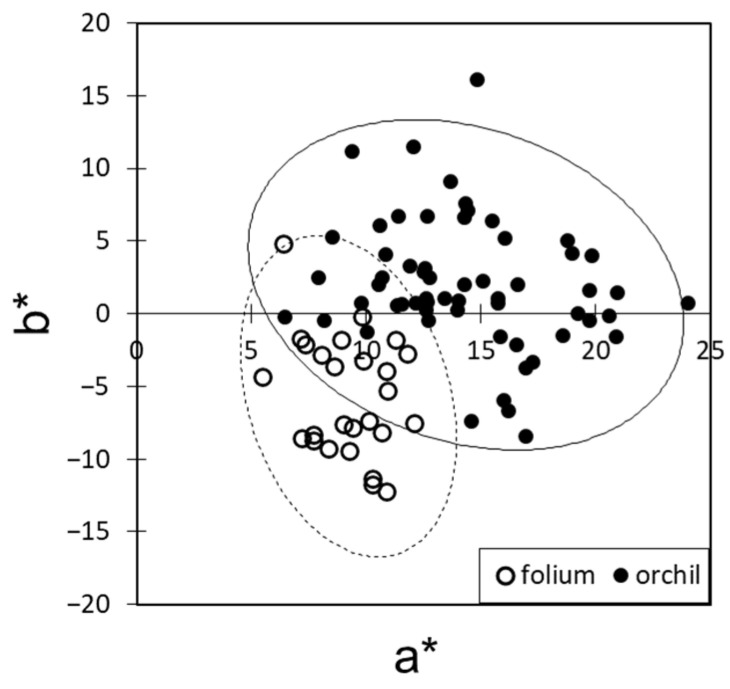
a* − b* chromatic distribution of orchil- and folium-dyed samples derived from HSI data. Orchil samples (black circles) are mainly distributed in the positive b* region, whereas folium samples (white circles) cluster in the negative b* domain. Confidence ellipses highlight the broader chromatic variability of orchil compared to the more compact distribution of folium.

This statistical evidence is fully consistent with the distribution observed in the a* − b* biplot (Figure 5), where confidence ellipses highlight a clear spatial separation mainly along the b* axis. Orchil samples are distributed over a broader and predominantly positive b* domain, reflecting their higher chromatic variability, likely related to the sensitivity of lichen-derived dyes to factors such as pH and preparation conditions. In contrast, folium samples form a more compact and well-defined cluster in the negative b* region, indicating greater chromatic homogeneity.

**Figure 6:**
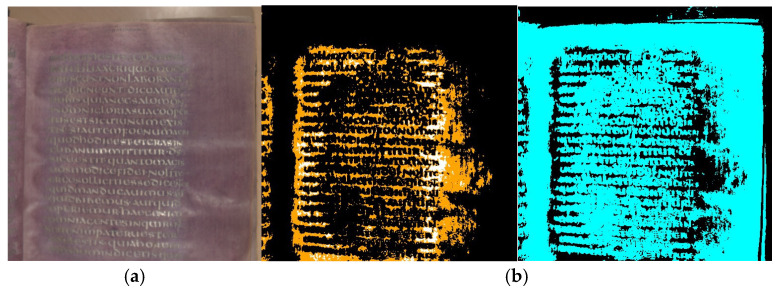
*Codex Brixianus* (BRIX). RGB image (**a**) and threshold-based mapping of a mixed purple folio derived from the hyperspectral b* map. (**a**) Map highlighting pixels within the folium-compatible and intermediate chromatic range. (**b**) Map showing areas characterized by orchil-like b* values. The spatial distribution of the two domains reveals significant chromatic heterogeneity across the folio, with the coexistence of distinct and transitional regions, reflecting the combined influence of dye composition, substrate interaction, and degradation processes. All maps were obtained from HSI data and visualized using fixed lookup tables (LUTs) to ensure consistency and comparability.

The spatial distribution of areas compatible with folium- and orchil-like chromatic behavior within the same folio was then investigated by mapping b* variability in the HSI-derived dataset. For the discrimination between orchil- and folium-dyed areas, threshold-based classification was applied to the b* coordinate, identified as the most effective discriminating parameter. Threshold values were defined from the statistical distributions of the reference datasets, using the interquartile ranges (IQR) of the two populations. In particular, the upper quartile of the folium distribution (Q3 = −2.61) and the lower quartile of the orchil distribution (Q1 = −0.16) were used to define conservative boundary values, minimizing misclassification between the two groups. Pixels with b* values lower than the folium Q3 were classified as folium-like, while values higher than the orchil Q1 were considered orchil-like. Intermediate values were treated as uncertain, reflecting the partial overlap between the two chromatic populations and the influence of substrate effects, degradation processes, and measurement variability. Figure 6 shows the threshold-based map of a folio from BRIX. Figure 6a discriminates folium-related areas characterized by b* < 3 (white) and −3 < b* < 0 (yellow) ranges, the latter being an intermediate class accounting for the partial overlap between the two chromatic populations of orchil and folium. Conversely, Figure 6b shows the distribution of orchil-based dye associated with b* > 0, which appears spatially differentiated and unevenly distributed across the page. The presence of the two dyes was also confirmed by FORS.

These maps indicate that b* is a useful parameter for chromatic segmentation in purple-dyed manuscripts, while also highlighting the limitations of single-parameter classification. Intermediate values cannot be unambiguously assigned and should be interpreted with caution, especially considering the systematic bias affecting b*. Therefore, b*-based mapping is best used as a screening and visualization tool, to be complemented by a* − b* analysis and spectral data.

#### 4.3.3. Monitoring Degradation: Abrasion and Photodegradation

The spatially resolved nature of hyperspectral imaging (HSI) makes it particularly suitable for detecting subtle chromatic variations that can support a more comprehensive assessment of degradation processes.

Spatially resolved a* and b* maps can particularly support the visualization of chromatic alterations associated with mechanical abrasion or color photodegradation.

**Figure 7:**
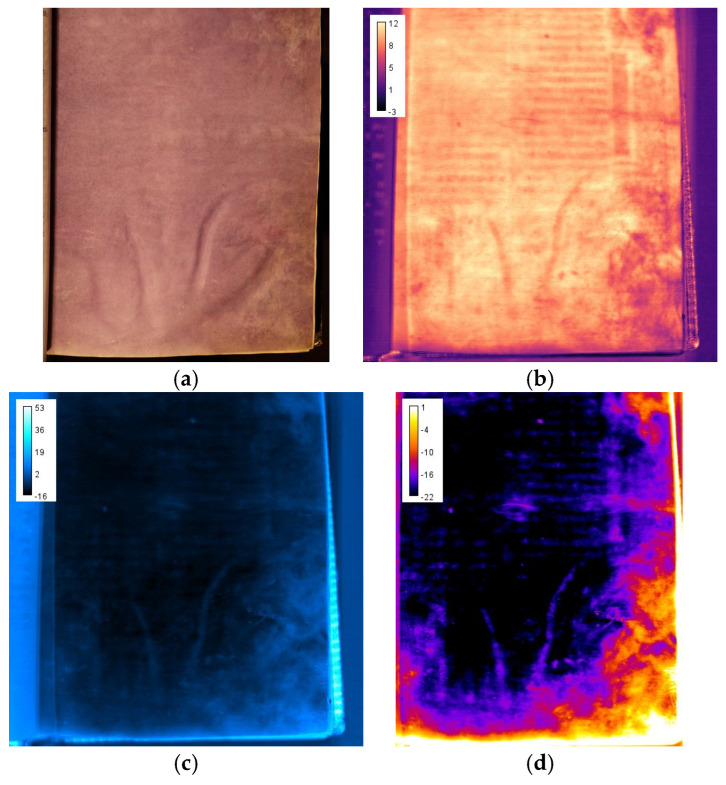
(**a**) High-resolution image of folio 10r from the *Breviario di Ferrante d’Aragona* (IB57). Spatial distributions of a*, b* maps derived from HSI data variations in the red–magenta component (**b**) and in the yellow–blue component (**c**) are also shown. The color scales represent the full range of measured a* or b* values. (**d**) (b* − a*) map obtained by combining the two datasets, enhancing areas characterized by increased substrate contribution and reduced intensity of the dye. All maps were obtained from HSI data and visualized using fixed lookup tables (LUTs) to ensure consistency and comparability. The color scale is centered on low (negative) to high (positive) index values, where higher values correspond to regions affected by partial dye loss and a stronger optical contribution of the parchment substrate.

Color variations associated with dye loss due to handling were investigated on a folium-dyed folio from IB57 (Figure 7a). The use of a fixed LUT scale ensured consistency and comparability across samples.

HSI-derived maps of color coordinates (Figure 7b,c) show that abraded areas systematically exhibited lower a* values and higher b* values compared to adjacent intact regions, as it could be expected following the partial removal of the purple dye layer and the increased optical contribution of the underlying parchment substrate.

In this way, by combining the trends observed along the a* and b* axes, the transition from dye-dominated to substrate-dominated optical behavior can be traced. The (b* − a*) map (Figure 7d) was obtained through a simple mathematical operation between TIFF images.

This approach enables the spatial identification and semi-quantitative assessment of abrasion patterns, enhancing subtle chromatic variations that may not be detectable through point-based measurements. It should be noted that the (b* − a*) map is not intended as a quantitative measure of degradation, but rather as a qualitative visualization tool that highlight relative chromatic differences. Its validity is supported by the spatial correspondence observed between areas identified as degraded in the visible image and regions characterized by higher index values, indicating patterns consistent with dye loss.

The effects of photodegradation were also investigated through differential chromatic mapping. The (Δb* − Δa*) maps highlight deviations from an “initial” original chromatic state, allowing the visualization of subtle color changes associated with photo-induced degradation. In particular, systematic decreases in a* values indicate a loss of red/purple chromatic components, while increases in b* values reflect a shift toward yellowish tones, typically related to the progressive exposure of the parchment substrate or alteration of the dye.

**Figure 8:**
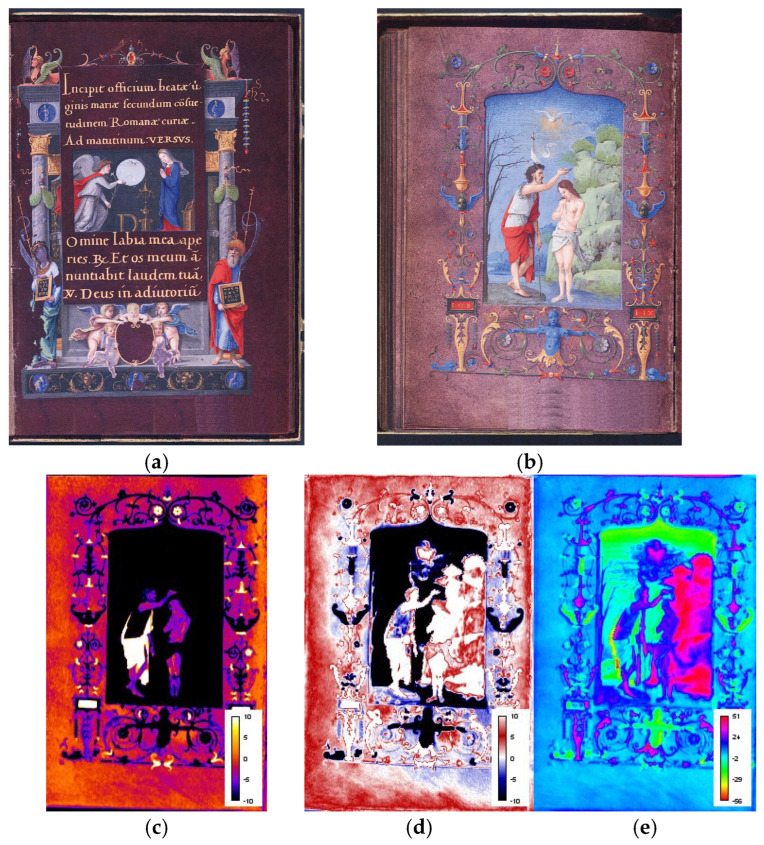
(**a**,**b**) RGB images of two folios from the Durazzo manuscript showing a well-preserved folio (15r) and a with a folio with visible photodegradation (167v). (**c**,**d**) Differential chromatic maps of folio 167v showing Δa* (**c**), Δb* (**d**), and the combined index (Δb* − Δa*) (**e**), calculated with respect to the average a* and b* values obtained from a set of 20 areas acquired on well preserved folios of the same codex. The maps reveal a consistent pattern of negative Δa* and positive Δb* values in the faded folio, indicating a progressive loss of the red–magenta component of the dye and an increased contribution of the yellow–brown parchment substrate. The combined index enhances the spatial visualization of photodegradation effects across the page.

Figure 8a,b show the effects of photodegradation on folio 167v from DUR, where repeated and prolonged light exposure during previous exhibitions has resulted in noticeable color fading. To evaluate this effect, reference chromatic values were derived from a representative set of 20 areas (10 × 10 pixels) selected from well-preserved folios (2r, 15r, 68r, 87r, 110v, and 207v). These areas showed relatively consistent a* values (17.9 ± 2.6) and more dispersed b* values (−3.7 ± 2.9), although remaining in the negative domain, and allowed the calculation of mean reference values, which were used as a baseline. Differential maps (Δa* and Δb*) were then obtained by computing pixel-wise deviations of folio 167v from the reference values.

The differential chromatic maps shown in Figure 8c–e provide insight into the spatial distribution of photodegradation across the analyzed folio. The Δa* map (Figure 8c) is dominated by negative values, indicating a systematic reduction of the red–magenta component associated with the purple dye. This trend is consistent with the progressive degradation of the chromophore system under light exposure, leading to a loss of saturation rather than a simple hue shift. Conversely, the Δb* map (Figure 8d) shows predominantly positive values, reflecting an increased contribution of the yellow–brown component of the parchment substrate.

When Δb* and Δa* maps are combined (Figure 8e), a spatial representation of the photo-degradation pattern is obtained. Areas exhibiting higher (Δb* − Δa*) values correspond to zones of more advanced degradation. The spatial heterogeneity of color variations across the page further suggests that photodegradation is controlled by local factors, including dye concentration, parchment microstructure, and exposure conditions.

Since photodegradation in purple-dyed manuscripts should be interpreted primarily as a desaturation process driven by the weakening of the dye and the concurrent increase in substrate contribution [27,49], differential chromatic mapping provides an effective tool for visualizing and comparing degradation patterns. This approach supports diagnostic interpretation and conservation practice by enabling early detection of chromatic alterations, informing exhibition strategies, and facilitating the monitoring of changes over time.

## 5. Conclusions

This study provides a systematic evaluation of the colorimetric performance of portable hyperspectral imaging (HSI) applied to complex surfaces, with specific focus on purple-dyed manuscripts.

Under controlled laboratory conditions (Set 1), HSI showed good agreement with spectrocolorimetric measurements under optimized acquisition settings, with mean ΔE_00_ values generally below 2. Nevertheless, systematic biases were observed, particularly a positive offset in a* and a negative bias in b*. The optimization of acquisition parameters—especially the use of custom white calibration and low-reflectance backgrounds—proved essential to reduce variability and improve repeatability.

In contrast, in situ measurements (Set 2) exhibited a marked reduction in accuracy, with an average ΔE_00_ of 4.32 ± 2.34 and increased dispersion across all colorimetric coordinates. These deviations were associated with material-related factors, including surface heterogeneity, non-planarity, and degradation phenomena, which significantly affect lightness (ΔL*) and increase variability in the b* coordinate.

Despite these limitations, HSI was demonstrated to preserve stable relative chromatic relationships in the a* − b* space, enabling reliable comparative analysis. This was demonstrated by the consistent separation between orchil- and folium-dyed samples, primarily along the b* coordinate, and by the preservation of chromatic trends related to substrate properties (e.g., hair vs. flesh side) and degradation processes.

The application to purple codices highlights the diagnostic potential of HSI. Spatial mapping of a* and b* coordinates enabled the visualization of chromatic heterogeneity, dye distribution, and degradation patterns. In particular, the use of empirical descriptors such as (b* − a*) and (Δb* − Δa*) proved effective for enhancing substrate contribution and identifying areas affected by mechanical abrasion and photodegradation. However, these descriptors should be regarded primarily as screening tools for visualizing degradation-related trends rather than as quantitative diagnostic parameters.

Overall, while HSI cannot be considered a fully reliable tool for absolute colorimetric measurements under in s.itu conditions, it represents a robust and powerful method for spatially resolved and comparative color analysis, with significant potential for diagnostic and conservation applications.

## Figures and Tables

**Table 1:** Overview of the purple codices analysed in the present study.

ID	Manuscript	Shelfmark	Institution	City, Country	Date (Century CE)	Purple Dye	Conservation State
LAT-3	*Codex Neapolitanus*	Lat. 3, ex vindob. Lat. 1235	BNC	Naples, IT	5th	Orchil	Good
BRIX	*Codex Brixianus*	F or VL10	Biblioteca Queriniana	Brescia, IT	6th	Orchil/folium	Moderate
SARZ	*Codex Purpureus Sarzanensis*	j or 22	Museo Diocesano d’Arte Sacra	Tortona, IT	5–6th	Orchil	Poor
GR-2	*Evangeliarium*	Gr. 2, ex vindob. Gr. Suppl. 12	BNC	Naples, IT	10th	Orchil	Good
HERC	*Ercole Senofontio*	BP.1099	Biblioteca Civica	Padua, IT	15th	Orchil	Good
DUR	*Horae Beatae Mariae Virginis*, *or Durazzo Book of Hours*	m.r. Cf. Arm. 1	Biblioteca Civica Berio	Genoa, IT	16th	Orchil	Excellent
IB57	*Breviario di Ferrante d’Aragona*	I.B.57	BNC	Naples, IT	15th	Folium	Good
XIX27	*Book of Hours*	XIX.27	BNC	Naples, IT	15th	Folium	Good
XII34	*Plutarco*, *Heroum clarissimorum-que virorum divinae*	XII.E.34	BNC	Naples, IT	15th	Folium	Good
ARTE	*Registro delle Matricole dell’Arte della Seta*		Archivio di Stato	Naples, IT	16th	Folium	Good

BNC = Biblioteca Nazionale Centrale Vittorio Emanuele III.

**Table 2:** Resume of Acquisition conditions.

ID	Background Color	White Reference Acquisition Mode
**B-CS**	Black	Custom
**Gy-CS**	Gray	Custom
**W-CS**	White	Custom
**LY-CS**	Light Yellow	Custom
**B-SM**	Black	Simultaneous
**Gy-SM**	Gray	Simultaneous
**W-SM**	White	Simultaneous
**LY-SM**	Light Yellow	Simultaneous

**Table 3:** Summary of inter-instrument differences between HSI-derived and spectrocolorimetric measurements (Konica Minolta CM-700d) under different experimental setups. Results are reported in terms of ΔE_00_, ΔL*, Δa*, and Δb* (HSI—colorimeter) for SCI and SCE geometries, considering different background conditions (black, gray, white, and light yellow) and white reference calibration modes (CS: custom; SM: simultaneous).

White Reference Calibration (HSI)	CS								SM							
Measurement Geometry (Colorimeter)	SCI				SCE				SCI				SCE			
	ΔE_00_ _inter_	ΔL* _inter_	Δa* _inter_	Δb* _inter_	ΔE_00_ _inter_	ΔL* _inter_	Δa* _inter_	Δb* _inter_	ΔE_00_ _inter_	ΔL* _inter_	Δa* _inter_	Δb* _inter_	ΔE_00_ _inter_	ΔL* _inter_	Δa* _inter_	Δb* _inter_
**Experimental set up**	**B-CS**								**B-SM**							
Mean	1.41	1.55	0.42	−0.64	1.45	1.60	0.43	−0.69	1.96	2.25	0.10	−0.63	2.00	2.31	0.12	−0.68
Median	1.43	1.59	0.54	−0.58	1.45	1.62	0.58	−0.59	1.90	2.24	0.32	−0.52	1.96	2.21	0.41	−0.49
SD	0.42	0.54	0.93	0.60	0.42	0.54	0.95	0.61	0.30	0.50	1.06	0.75	0.31	0.50	1.08	0.77
RMSE		1.64	1.02	0.88		1.69	1.05	0.92		2.31	1.06	0.98		2.36	1.09	1.03
**Experimental set up**	**Gy-CS**								**Gy-SM**							
Mean	1.36	1.10	0.29	−1.63	1.39	1.15	0.30	−1.67	2.35	2.51	0.23	−1.87	2.39	2.56	0.24	−1.91
Median	1.19	1.32	0.52	−0.89	1.18	1.33	0.52	−0.92	2.28	2.66	0.25	−1.33	2.32	2.63	0.34	−1.43
SD	0.58	0.35	0.59	1.77	0.59	0.34	0.60	1.72	0.49	0.46	0.78	1.17	0.50	0.46	0.80	1.13
RMSE		1.15	0.66	2.41		1.20	0.67	2.40		2.55	0.82	2.21		2.60	0.84	2.23
**Experimental set up**	**W-CS**								**W-SM**							
Mean	2.16	1.62	0.73	−3.10	2.20	1.67	0.74	−3.14	3.15	2.67	0.65	−4.26	2.82	2.58	0.66	−3.46
Median	1.83	1.73	0.63	−2.83	1.86	1.82	0.66	−2.85	2.65	2.88	0.44	−4.06	2.42	2.64	0.80	−3.17
SD	0.92	0.40	0.60	2.34	0.93	0.42	0.60	2.30	1.03	0.37	0.63	2.63	0.86	0.44	0.65	1.94
RMSE		1.67	0.95	3.89		1.72	0.96	3.89		2.70	0.91	5.01		2.62	0.93	3.97
**Experimental set up**	**LY-CS**								**LY-SM**							
Mean	2.76	1.97	0.80	−4.32	2.79	2.02	0.82	−4.36	3.15	2.67	0.65	−4.26	3.19	2.73	0.66	−4.30
Median	2.48	2.07	0.78	−4.09	2.50	2.17	0.78	−4.11	2.65	2.88	0.44	−4.06	2.68	2.98	0.46	−4.09
SD	1.15	0.44	0.63	2.87	1.16	0.45	0.63	2.82	1.03	0.37	0.63	2.63	1.04	0.38	0.64	2.58
RMSE		2.01	1.02	5.19		2.07	1.03	5.20		2.70	0.91	5.01		2.75	0.92	5.02

CS = Custom; SM = Simultaneous.

**Table 4:** Distribution of inter-instrumental ΔE_00_ values (%) across perceptual tolerance thresholds under different acquisition conditions.

Perceptual Classification	Measurement Geometry (Colorimeter)	SCE		SCI	
	White Reference Calibration (HSI)	CS	SM	CS	SM
Indistinguishable	ΔE_00inter_ ≤ 1	13	0	13	0
Acceptable	1 < ΔE_00inter_ ≤ 2	47	16	53	16
Perceptible	2 < ΔE_00inter_ ≤ 3.5	31	69	25	63
Critical	3.5 < ΔE_00inter_ ≤ 5	6	13	6	16
Evident	ΔE_00inter_ > 5	3	3	3	6
**TOTAL**		100	100	100	100

**Table 5:** Comparison of colorimetric data of Set 2 obtained via HSI (custom white—black background) compared with reference data obtained via Konica-Minolta colorimeter in SCE mode reported as ΔE_00inter_, ΔL*_inter_, Δa*_inter_ and Δb*_inter_.

Codex ID	ΔE_00inter_	ΔL*_inter_	Δa*_inter_	Δb*_inter_
LAT-3	4.17	−0.40	3.28	−2.77
LAT-3	4.20	0.02	4.66	−3.23
LAT-3	2.21	−1.51	2.63	0.38
LAT-3	2.99	−1.85	2.22	−1.87
LAT-3	3.89	−1.66	3.90	−1.37
LAT-3	3.68	−0.16	4.47	−1.55
LAT-3	2.75	−0.34	3.80	−1.79
LAT-3	2.67	−0.28	3.29	−0.33
LAT-3	2.20	0.83	2.49	−0.69
LAT-3	0.98	1.11	−0.01	−0.24
LAT-3	3.68	1.30	4.46	−0.22
LAT-3	4.20	−1.41	4.40	−1.02
LAT-3	3.15	−2.62	2.30	−0.61
LAT-3	2.40	−0.14	3.24	−0.40
LAT-3	3.79	−1.46	3.03	−2.45
LAT-3	2.69	−0.34	2.50	−0.94
DUR	4.17	−0.57	0.12	−6.10
DUR	3.98	−4.29	3.01	−0.46
DUR	4.05	−4.05	1.79	−3.63
DUR	4.11	0.08	2.74	−5.14
SARZ	5.24	−4.48	−0.06	−3.85
SARZ	4.94	−5.01	−1.54	−2.34
SARZ	8.69	−7.96	2.61	−6.89
SARZ	7.99	−5.26	2.22	−8.17
SARZ	9.77	−9.10	2.96	−5.55
SARZ	11.09	−11.52	0.89	−4.72
GR-2	3.03	−0.47	2.47	−1.91
**Mean**	4.32	−2.28	2.51	−2.51
**Median**	3.89	−1.41	2.63	−1.87
**SD**	2.34	3.16	1.50	2.24
**R^2^**		0.92	0.81	0.87
**RMSE**		3.90	2.92	3.37

**Table 6:** HSI-derived CIELAB coordinates (L*, a*, b*) for selected areas of purple codices dyed with orchil or folium.

Codex ID	L	a	b	Dye
LAT-3	59.83	11.29	4.36	Orchil
LAT-3	48.55	19.79	4.07	Orchil
LAT-3	46.72	20.57	−0.14	Orchil
LAT-3	59.28	11.31	0.59	Orchil
LAT-3	47.83	12.69	−0.42	Orchil
LAT-4	43.44	15.72	0.79	Orchil
LAT-3	43.90	20.90	1.52	Orchil
LAT-3	52.77	14.26	2.00	Orchil
LAT-3	55.82	13.99	0.96	Orchil
LAT-3	39.65	19.70	1.61	Orchil
LAT-3	50.59	15.72	1.10	Orchil
LAT-3	50.81	12.64	0.86	Orchil
LAT-3	60.68	10.53	2.05	Orchil
LAT-3	54.59	16.58	2.04	Orchil
LAT-3	52.56	14.38	7.16	Orchil
LAT-3	58.82	10.56	6.13	Orchil
DUR	32.89	16.94	−3.71	Orchil
DUR	27.96	16.51	−2.08	Orchil
DUR	26.01	15.96	−5.90	Orchil
DUR	33.60	19.19	0.05	Orchil
DUR	26.28	16.94	−8.40	Orchil
DUR	38.24	14.55	−7.37	Orchil
DUR	34.14	17.21	−3.33	Orchil
DUR	26.23	20.88	−1.57	Orchil
DUR	24.80	24.00	0.77	Orchil
DUR	33.56	19.70	−0.44	Orchil
DUR	34.49	18.52	−1.47	Orchil
DUR	39.46	16.16	−6.62	Orchil
SARZ	46.02	13.62	9.13	Orchil
SARZ	40.81	14.31	7.61	Orchil
SARZ	28.35	16.04	5.23	Orchil
SARZ	30.90	15.48	6.40	Orchil
SARZ	38.12	14.82	16.16	Orchil
SARZ	36.35	12.01	11.48	Orchil
GR-2	27.39	8.14	−0.41	Orchil
GR-2	46.43	11.35	6.75	Orchil
GR-2	30.86	6.44	−0.16	Orchil
GR-2	45.33	12.47	2.92	Orchil
GR-2	46.87	8.51	5.34	Orchil
GR-2	38.67	9.78	0.77	Orchil
GR-2	43.34	9.35	11.21	Orchil
GR-2	38.62	10.84	4.11	Orchil
GR-2	33.65	10.69	2.49	Orchil
HERC	33.99	12.51	3.12	Orchil
HERC	24.01	12.75	2.53	Orchil
HERC	30.37	11.90	3.29	Orchil
HERC	24.35	15.80	−1.55	Orchil
HERC	22.31	13.96	0.30	Orchil
HERC	22.06	10.02	−1.26	Orchil
HERC	41.86	14.24	6.65	Orchil
HERC	44.73	12.64	6.71	Orchil
HERC	31.89	15.05	2.32	Orchil
BRIX	36.84	7.89	2.48	Orchil
BRIX	35.89	18.96	4.19	Orchil
BRIX	36.59	18.72	5.07	Orchil
BRIX	38.58	11.50	0.71	Orchil
BRIX	36.54	12.57	1.05	Orchil
BRIX	42.39	12.57	0.30	Folium
BRIX	45.01	12.11	0.76	Folium
BRIX	43.61	13.38	1.12	Folium
ARTE	48.08	12.10	−7.57	Folium
ARTE	52.05	11.78	−2.77	Folium
ARTE	57.05	9.84	−0.23	Folium
ARTE	56.49	6.39	4.80	Folium
XIX27	50.55	9.90	−3.25	Folium
XIX27	60.11	8.62	−3.64	Folium
XIX27	47.98	7.14	−1.76	Folium
XIX 27	45.41	7.36	−2.13	Folium
XIX 27	60.36	5.50	−4.38	Folium
XIX 27	60.45	11.32	−1.84	Folium
XIX 27	48.98	10.91	−3.97	Folium
XIX 27	53.66	10.94	−5.33	Folium
IB57	33.05	10.31	−11.40	Folium
IB57	36.09	10.91	−12.23	Folium
IB57	44.03	10.27	−11.75	Folium
IB57	52.04	8.40	−9.29	Folium
IB57	49.16	9.28	−9.51	Folium
IB57	37.16	7.20	−8.56	Folium
IB57	40.15	7.71	−8.35	Folium
IB57	37.27	7.74	−8.74	Folium
XII34	38.71	10.72	−8.20	Folium
XII34	39.46	10.14	−7.43	Folium
XII34	36.38	9.45	−7.87	Folium
XII34	43.57	8.07	−2.85	Folium
XII34	40.67	9.04	−7.63	Folium
XII34	44.66	8.91	−1.83	Folium

**Table 7:** Comparison of CIELAB colorimetric parameters (L*, a*, b*) for orchil- and folium-dyed samples. Median values and interquartile ranges (IQR) are reported for each group. Statistical differences were evaluated using the Mann–Whitney U test; U and Z statistics, *p*-values, and effect sizes (r) are provided. Significant differences are observed for all parameters, with the strongest discrimination along the b* axis, confirming its key role in distinguishing between orchil (positive b* values) and folium (negative b* values).

	Orchil		Folium					
Parameter	Median	IQR	Median	IQR	U	Z	*p*-Value	Effect Size (r)
L*	38.60	14.18	46.69	13.18	451	−3.09	0.0020	−0.33
a*	14.12	4.96	9.36	3.03	1408	5.90	<0.0001	0.63
b*	1.32	4.47	−6.38	5.99	1432	6.13	<0.0001	0.66

## Data Availability

Representative sample datasets supporting the findings of this study, including examples of data at different processing stages, are publicly available in the Zenodo repository, DOI: 10.5281/zenodo.20322170.

## Supplementary Materials

The following supporting information can be downloaded at: https://www.mdpi.com/article/10.3390/s26113358/s1, Figure S1: Overview of selected folios dyed with orchil. The images show recto and verso of representative folios from GR-2 (a,b), LAT-3 (c,d), and DUR (e,f) manuscripts; Figure S2: Overview of selected folios dyed with folium. The images show recto and verso of representative folios from IB57 (a,b), XIX27 (c,d), and XII34 (e,f) manuscripts; Table S1: Colorimetric data acquired on Diffuse Reflectance Spectralon^®^ Color Standards (Labsphere, New Hampshire, USA) using a Konica–Minolta colorimeter, with a 2° standard observer and D65 illuminant, SCI/SCE mode. Table S2—Colorimetric data obtained via HSI with different setup parameters. Mean values are reported for color coordinates. ΔE00, ΔL*, Δa* and Δb* are calculated from reference data obtained via Konica-Minolta colorimeter in SCI mode and SCE mode.
