# Supplementary material for: Hyperspectral Imaging for the Colorimetric Characterization of Purple Manuscripts: Accuracy, Biases, and Diagnostic Potential
Source: Sensors (Basel). 2026 May 26;26(11):3358. doi: 10.3390/s26113358 (PMC13259110; doi:10.3390/s26113358)
Supplement: Supplementary file 1 [file sensors-26-03358-s001.zip › sensors-4264472-supplementary.pdf]

Figure S1. Overview of selected folios dyed with orchil. The images show recto and verso of representative folios from GR-2 (a-b), LAT-3 (c-d), and DUR (e-f) manuscripts.

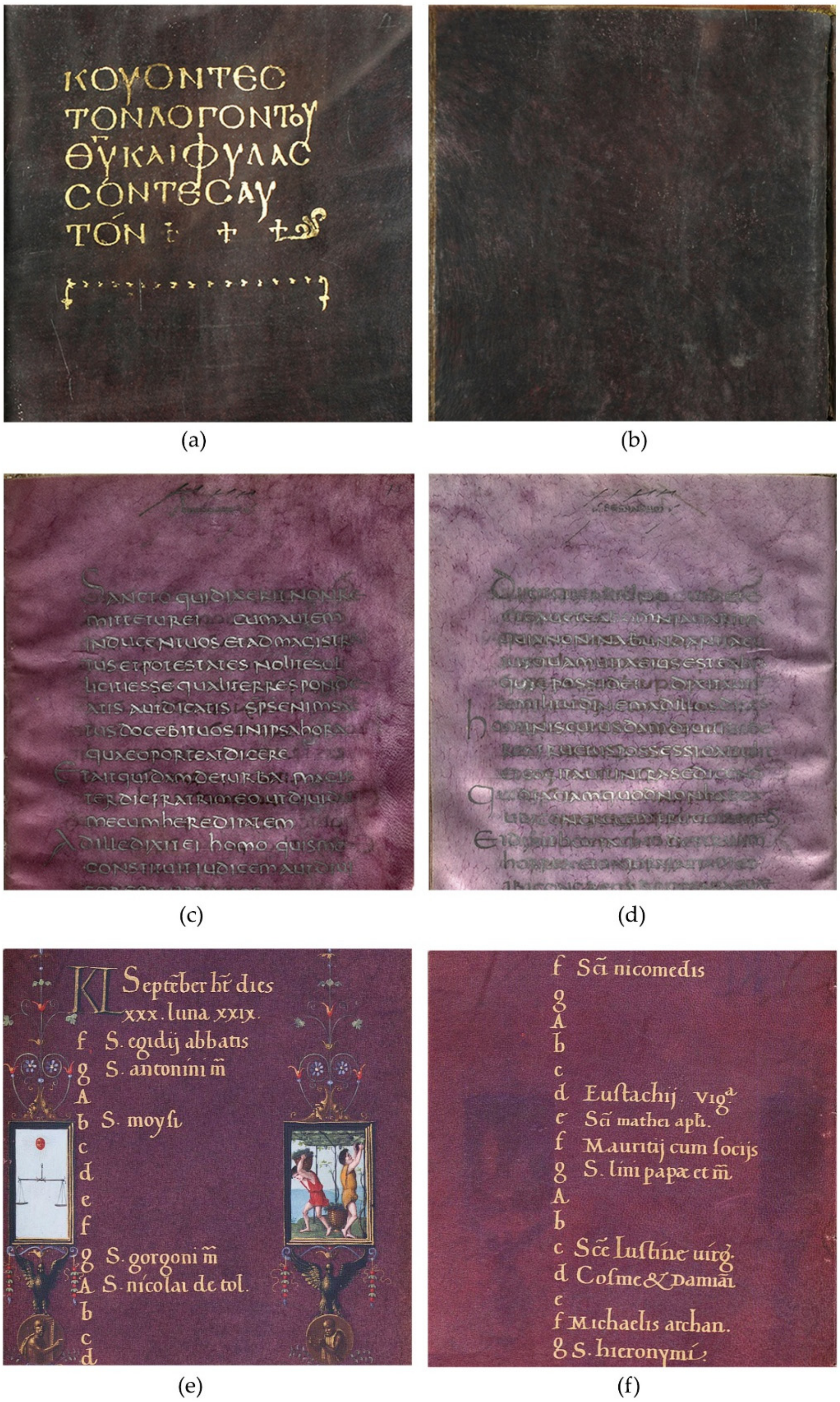

Figure S2. Overview of selected folios dyed with folium. The images show recto and verso of representative folios from IB57 (a-b), XIX27 (c-d), and XII34 (e-f) manuscripts

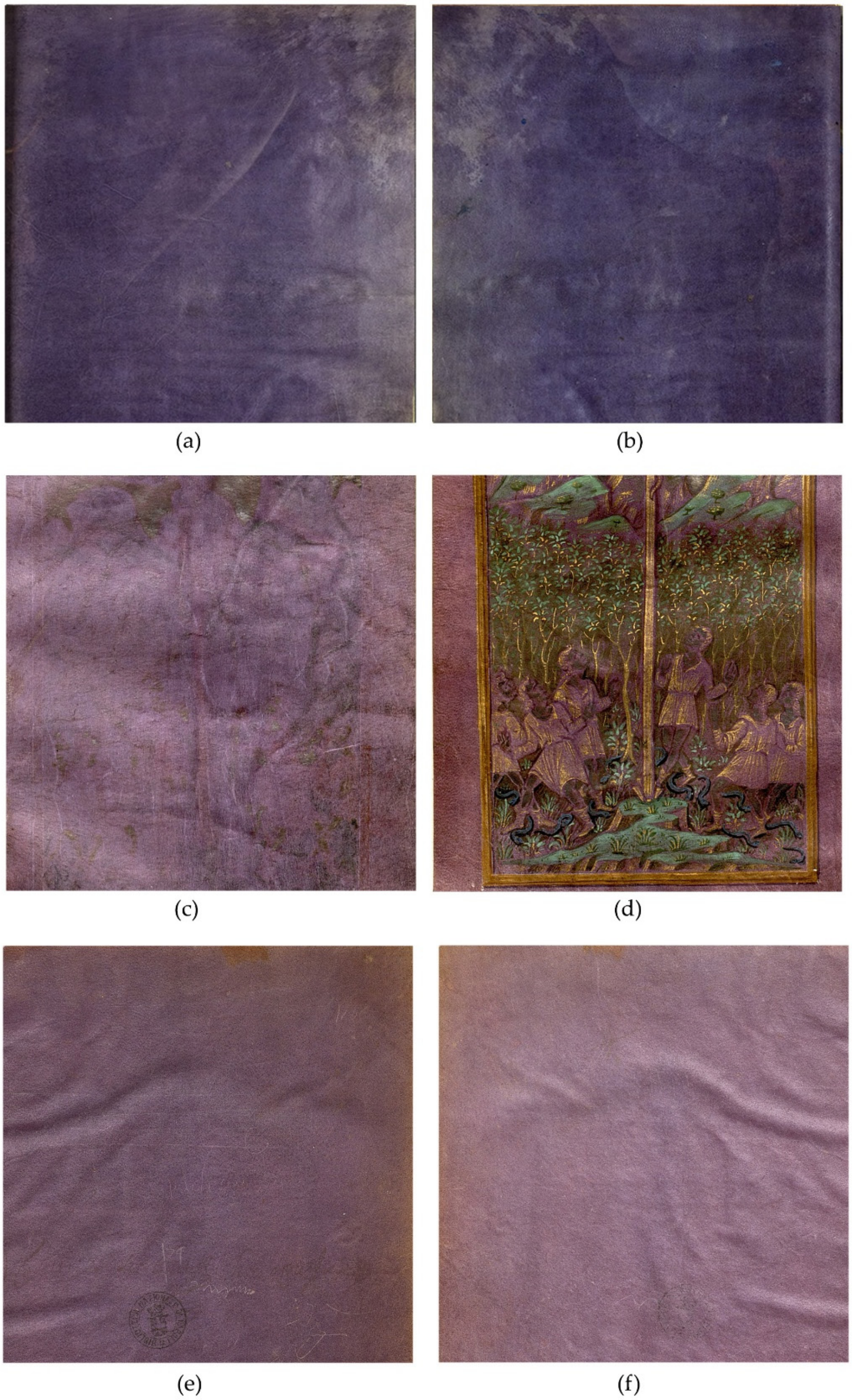

Table S1 – Colorimetric data acquired on Diffuse Reflectance Spectralon® Color Standards (Labsphere, New Hampshire, USA) using a Konica–Minolta colorimeter, with a 2° standard observer and D65 illuminant, SCI/SCE configuration mode.

**Color Standards**		**SCI**			**SCE**		
**ID**	**Color**	**L^*_{COL}**	**a^*_{COL}**	**b^*_{COL}**	**L^*_{COL}**	**a^*_{COL}**	**b^*_{COL}**
SCS-BL-010	blue	57.81	2.98	-43.05	57.77	2.96	-42.95
SCS-GN-010	green	63.00	-30.61	15.53	62.96	-30.61	15.56
SCS-VI-010	violet	64.01	18.40	-22.21	64.04	18.31	-22.05
SCS-YW-010	yellow	87.69	2.59	85.01	87.59	2.62	84.97
SCS-RD-010	red	49.97	51.16	24.28	49.88	51.14	24.32
SCS-OR-010	orange	69.48	45.42	40.76	69.46	45.34	40.73
SCS-CY-010	cyan	72.07	-28.08	-11.39	72.00	-28.03	-11.34
SCS-PU-010	purple	44.41	14.93	-4.96	44.32	14.95	-4.94

Table S2 – Colorimetric data obtained via HSI with different setup parameters. Mean values are reported for colour coordinates. ΔE00, ΔL*, Δa* and Δb* are calculated from reference data obtained via Konica-Minolta colorimeter in SCI mode and SCE mode.

Set up: B-CS										SCI				SCE			
**Color**	**L*$_{HSI}$**			**a*$_{HSI}$**			**b*$_{HSI}$**			**ΔE00$_{inter}$**	**ΔL*$_{inter}$**	**Δa*$_{inter}$**	**Δb*$_{inter}$**	**ΔE00$_{inter}$**	**ΔL*$_{inter}$**	**Δa*$_{inter}$**	**Δb*$_{inter}$**
blue	**59.10**	±	0.26	**3.11**	±	0.13	**-44.50**	±	0.26	1.25	1.29	0.13	-1.45	1.29	1.33	0.15	-1.55
green	**63.86**	±	0.26	**-31.18**	±	0.22	**15.35**	±	0.19	0.78	0.86	-0.57	-0.18	0.81	0.90	-0.57	-0.21
violet	**65.62**	±	0.26	**18.94**	±	0.27	**-22.21**	±	0.30	1.37	1.60	0.54	0.00	1.35	1.57	0.63	-0.16
yellow	**89.87**	±	0.24	**3.60**	±	0.14	**85.03**	±	0.48	1.49	2.18	1.01	0.02	1.55	2.28	0.98	0.06
red	**51.55**	±	0.23	**52.69**	±	0.46	**22.58**	±	0.30	2.01	1.58	1.53	-1.70	2.10	1.67	1.55	-1.74
orange	**71.62**	±	0.27	**46.92**	±	0.36	**40.25**	±	0.51	1.86	2.14	1.50	-0.51	1.88	2.16	1.58	-0.48
cyan	**74.15**	±	0.24	**-29.41**	±	0.21	**-12.04**	±	0.17	1.67	2.08	-1.33	-0.65	1.73	2.15	-1.38	-0.70
purple	**45.08**	±	0.27	**15.48**	±	0.18	**-5.64**	±	0.25	0.84	0.67	0.55	-0.68	0.91	0.76	0.53	-0.70

Set up: Gy-CS										SCI				SCE			
**Color**	**L*$_{HSI}$**			**a*$_{HSI}$**			**b*$_{HSI}$**			**ΔE00$_{inter}$**	**ΔL*$_{inter}$**	**Δa*$_{inter}$**	**Δb*$_{inter}$**	**ΔE00$_{inter}$**	**ΔL*$_{inter}$**	**Δa*$_{inter}$**	**Δb*$_{inter}$**
blue	**58.74**	±	0.25	**2.65**	±	0.13	**-43.16**	±	0.30	0.88	0.93	-0.33	-0.11	0.92	0.97	-0.31	-0.21
green	**63.48**	±	0.22	**-30.09**	±	0.22	**14.64**	±	0.17	0.62	0.48	0.52	-0.89	0.65	0.52	0.52	-0.92
violet	**65.37**	±	0.35	**18.47**	±	0.33	**-21.63**	±	0.41	1.19	1.36	0.07	0.58	1.15	1.33	0.16	0.42
yellow	**88.80**	±	0.28	**3.84**	±	0.14	**80.57**	±	0.57	1.42	1.11	1.25	-4.44	1.44	1.21	1.22	-4.40
red	**51.29**	±	0.23	**51.69**	±	0.42	**20.38**	±	0.28	2.55	1.32	0.53	-3.90	2.62	1.41	0.55	-3.94
orange	**70.97**	±	0.29	**45.97**	±	0.41	**37.84**	±	0.53	1.99	1.49	0.55	-2.92	2.01	1.51	0.63	-2.89
cyan	**73.48**	±	0.23	**-28.91**	±	0.29	**-11.42**	±	0.13	1.12	1.41	-0.83	-0.03	1.18	1.48	-0.88	-0.08
purple	**45.10**	±	0.30	**15.45**	±	0.19	**-6.27**	±	0.26	1.11	0.69	0.52	-1.31	1.17	0.77	0.50	-1.33

Set up: W-CS										SCI				SCE			
**Color**	**L*$_{HSI}$**			**a*$_{HSI}$**			**b*$_{HSI}$**			**ΔE00$_{inter}$**	**ΔL*$_{inter}$**	**Δa*$_{inter}$**	**Δb*$_{inter}$**	**ΔE00$_{inter}$**	**ΔL*$_{inter}$**	**Δa*$_{inter}$**	**Δb*$_{inter}$**
blue	**59.46**	±	0.22	**3.61**	±	0.15	**-44.26**	±	0.29	1.50	1.65	0.63	-1.21	1.54	1.69	0.65	-1.31
green	**64.06**	±	0.24	**-28.75**	±	0.21	**11.40**	±	0.17	2.44	1.06	1.86	-4.13	2.46	1.10	1.86	-4.16
violet	**65.77**	±	0.27	**18.80**	±	0.25	**-22.87**	±	0.30	1.49	1.76	0.40	-0.65	1.49	1.72	0.49	-0.81
yellow	**89.76**	±	0.29	**4.19**	±	0.15	**76.43**	±	0.47	2.52	2.07	1.60	-8.58	2.54	2.17	1.57	-8.54
red	**52.43**	±	0.20	**51.25**	±	0.45	**16.12**	±	0.22	5.03	2.46	0.09	-8.17	5.09	2.55	0.11	-8.21
orange	**71.95**	±	0.29	**46.37**	±	0.42	**34.68**	±	0.59	3.85	2.47	0.95	-6.08	3.86	2.49	1.03	-6.05
cyan	**74.25**	±	0.18	**-28.13**	±	0.27	**-13.11**	±	0.17	1.95	2.18	-0.05	-1.72	2.01	2.25	-0.10	-1.77
purple	**46.49**	±	0.24	**15.86**	±	0.21	**-9.01**	±	0.23	3.29	2.08	0.93	-4.05	3.36	2.17	0.91	-4.07

**Set up: LY-CS**				**SCI**				**SCE**			
**Color**	$\mathbf{L^*_{HSI}}$	$\mathbf{a^*_{HSI}}$	$\mathbf{b^*_{HSI}}$	$\mathbf{\Delta E00_{inter}}$	$\mathbf{\Delta L^*_{inter}}$	$\mathbf{\Delta a^*_{inter}}$	$\mathbf{\Delta b^*_{inter}}$	$\mathbf{\Delta E00_{inter}}$	$\mathbf{\Delta L^*_{inter}}$	$\mathbf{\Delta a^*_{inter}}$	$\mathbf{\Delta b^*_{inter}}$
blue	**59.19** ± 0.24	**3.29** ± 0.14	**-43.66** ± 0.29	1.25	1.38	0.31	-0.61	1.29	1.42	0.33	-0.71
green	**63.72** ± 0.27	**-29.22** ± 0.24	**12.86** ± 0.16	1.57	0.72	1.39	-2.67	1.60	0.76	1.39	-2.70
violet	**65.52** ± 0.27	**18.81** ± 0.24	**-22.27** ± 0.28	1.27	1.51	0.41	-0.06	1.25	1.48	0.50	-0.22
yellow	**89.48** ± 0.22	**4.26** ± 0.14	**78.54** ± 0.53	2.10	1.79	1.67	-6.47	2.12	1.88	1.64	-6.43
red	**51.96** ± 0.21	**51.59** ± 0.47	**17.88** ± 0.25	4.02	1.99	0.43	-6.40	4.09	2.08	0.45	-6.44
orange	**71.54** ± 0.28	**46.51** ± 0.39	**36.22** ± 0.54	3.05	2.06	1.09	-4.54	3.07	2.08	1.17	-4.51
cyan	**73.91** ± 0.21	**-28.38** ± 0.22	**-12.48** ± 0.15	1.53	1.84	-0.30	-1.09	1.59	1.91	-0.35	-1.14
purple	**46.08** ± 0.24	**15.76** ± 0.16	**-7.94** ± 0.25	2.53	1.67	0.83	-2.98	2.59	1.76	0.81	-3.00

**Set up: B-SM**				**SCI**				**SCE**			
**Color**	$\mathbf{L^*_{HSI}}$	$\mathbf{a^*_{HSI}}$	$\mathbf{b^*_{HSI}}$	$\mathbf{\Delta E00_{inter}}$	$\mathbf{\Delta L^*_{inter}}$	$\mathbf{\Delta a^*_{inter}}$	$\mathbf{\Delta b^*_{inter}}$	$\mathbf{\Delta E00_{inter}}$	$\mathbf{\Delta L^*_{inter}}$	$\mathbf{\Delta a^*_{inter}}$	$\mathbf{\Delta b^*_{inter}}$
blue	**59.83** ± 0.23	**2.85** ± 0.12	**-44.88** ± 0.27	1.50	2.02	-0.13	-1.83	2.01	2.06	-0.11	-1.93
green	**64.48** ± 0.21	**-31.76** ± 0.18	**15.51** ± 0.14	2.44	1.48	-1.15	-0.02	1.37	1.52	-1.15	-0.05
violet	**66.25** ± 0.24	**18.72** ± 0.27	**-22.42** ± 0.32	1.49	2.24	0.32	-0.21	1.83	2.21	0.41	-0.37
yellow	**90.64** ± 0.24	**3.25** ± 0.11	**85.51** ± 0.43	2.52	2.95	0.66	0.50	1.96	3.05	0.63	0.54
red	**51.93** ± 0.21	**52.55** ± 0.43	**22.54** ± 0.31	5.03	1.96	1.39	-1.74	2.40	2.05	1.41	-1.78
orange	**72.23** ± 0.24	**46.81** ± 0.34	**40.24** ± 0.55	3.85	2.75	1.39	-0.52	2.28	2.77	1.47	-0.49
cyan	**74.89** ± 0.20	**-29.88** ± 0.25	**-12.05** ± 0.14	1.95	2.82	-1.80	-0.66	2.31	2.89	-1.85	-0.71
purple	**46.22** ± 0.24	**15.06** ± 0.16	**-5.54** ± 0.23	3.29	1.81	0.13	-0.58	1.85	1.90	0.11	-0.60

**Set up: Gy-SM**				**SCI**				**SCE**			
**Color**	$\mathbf{L^*_{HSI}}$	$\mathbf{a^*_{HSI}}$	$\mathbf{b^*_{HSI}}$	$\mathbf{\Delta E00_{inter}}$	$\mathbf{\Delta L^*_{inter}}$	$\mathbf{\Delta a^*_{inter}}$	$\mathbf{\Delta b^*_{inter}}$	$\mathbf{\Delta E00_{inter}}$	$\mathbf{\Delta L^*_{inter}}$	$\mathbf{\Delta a^*_{inter}}$	$\mathbf{\Delta b^*_{inter}}$
blue	**60.27** ± 0.27	**2.77** ± 0.13	-44.38 ± 0.26	2.28	2.46	-0.21	-1.33	2.32	2.50	-0.19	-1.43
green	**64.87** ± 0.20	**-30.93** ± 0.21	14.45 ± 0.17	1.70	1.87	-0.32	-1.08	1.73	1.91	-0.32	-1.11
violet	**66.67** ± 0.32	**18.65** ± 0.34	-22.52 ± 0.40	2.18	2.66	0.25	-0.31	2.17	2.63	0.34	-0.47
yellow	**90.58** ± 0.22	**3.44** ± 0.12	82.13 ± 0.49	1.99	2.89	0.85	-2.88	2.04	2.99	0.82	-2.84
red	**52.41** ± 0.24	**52.09** ± 0.44	20.16 ± 0.25	3.39	2.44	0.93	-4.12	3.47	2.53	0.95	-4.16
orange	**72.38** ± 0.27	**46.56** ± 0.36	38.24 ± 0.46	2.75	2.90	1.14	-2.53	2.77	2.92	1.22	-2.50
cyan	**75.19** ± 0.19	**-29.46** ± 0.27	-12.21 ± 0.15	2.41	3.12	-1.38	-0.82	2.47	3.19	-1.43	-0.87
purple	**46.14** ± 0.27	**15.52** ± 0.18	-6.89 ± 0.24	2.09	1.73	0.59	-1.93	2.17	1.82	0.57	-1.95

Set up: W-SM									SCI				SCE			
**Color**	**L^*_{HSI}**			**a^*_{HSI}**			**b^*_{HSI}**		**$\Delta E00_{inter}$**	**ΔL^*_{inter}**	**Δa^*_{inter}**	**Δb^*_{inter}**	**$\Delta E00_{inter}$**	**ΔL^*_{inter}**	**Δa^*_{inter}**	**Δb^*_{inter}**
blue	**60.39**	±	0.22	**3.42**	± 0.13		**-44.57**	± 0.27	2.32	2.58	0.44	-1.52	2.36	2.62	0.46	-1.62
green	**64.86**	±	0.24	**-29.16**	± 0.23		**11.47**	± 0.17	2.71	1.86	1.45	-4.06	2.74	1.90	1.45	-4.09
violet	**66.46**	±	0.24	**18.67**	± 0.22		**-23.06**	± 0.26	2.06	2.45	0.27	-0.85	2.06	2.42	0.36	-1.01
yellow	**90.57**	±	0.23	**3.96**	± 0.12		**77.21**	± 0.51	2.65	2.88	1.37	-7.80	2.68	2.98	1.34	-7.76
red	**52.94**	±	0.18	**51.49**	± 0.40		**16.16**	± 0.25	5.30	2.97	0.33	-8.13	5.37	3.06	0.35	-8.17
orange	**72.50**	±	0.24	**46.54**	± 0.29		**34.97**	± 0.49	3.99	3.02	1.11	-5.79	4.01	3.04	1.19	-5.76
cyan	**75.05**	±	0.19	**-28.67**	± 0.19		**-13.15**	± 0.14	2.45	2.98	-0.59	-1.76	2.51	3.05	-0.64	-1.81
purple	**47.07**	±	0.23	**15.72**	± 0.14		**-9.14**	± 0.21	3.72	2.66	0.79	-4.18	3.79	2.75	0.77	-4.20

Set up: LY-SM									SCI				SCE			
**Color**	**L^*_{HSI}**			**a^*_{HSI}**			**b^*_{HSI}**		**$\Delta E00_{inter}$**	**ΔL^*_{inter}**	**Δa^*_{inter}**	**Δb^*_{inter}**	**$\Delta E00_{inter}$**	**ΔL^*_{inter}**	**Δa^*_{inter}**	**Δb^*_{inter}**
blue	**60.14**	±	0.26	**3.31**	± 0.15		**-44.50**	± 0.24	2.11	2.33	0.33	-1.45	2.15	2.37	0.35	-1.55
green	**64.63**	±	0.29	**-29.80**	± 0.24		**12.69**	± 0.17	2.05	1.63	0.81	-2.84	2.09	1.67	0.81	-2.87
violet	**66.48**	±	0.28	**18.85**	± 0.27		**-23.11**	± 0.31	2.08	2.47	0.45	-0.90	2.08	2.44	0.54	-1.06
yellow	**90.65**	±	0.24	**3.89**	± 0.14		**79.10**	± 0.54	2.41	2.96	1.30	-5.91	2.45	3.06	1.27	-5.87
red	**52.72**	±	0.21	**52.01**	± 0.44		**17.67**	± 0.26	4.58	2.75	0.85	-6.61	4.65	2.84	0.87	-6.65
orange	**72.47**	±	0.29	**46.83**	± 0.42		**36.20**	± 0.49	3.56	2.99	1.41	-4.56	3.58	3.01	1.49	-4.53
cyan	**74.92**	±	0.23	**-28.84**	± 0.24		**-13.01**	± 0.16	2.33	2.85	-0.76	-1.62	2.39	2.92	-0.81	-1.67
purple	**46.63**	±	0.25	**15.75**	± 0.19		**-8.42**	± 0.26	3.10	2.22	0.82	-3.46	3.17	2.31	0.80	-3.48
